# Superoxide Dismutase 1 in Health and Disease: How a Frontline Antioxidant Becomes Neurotoxic

**DOI:** 10.1002/anie.202000451

**Published:** 2020-11-19

**Authors:** Benjamin G. Trist, James B. Hilton, Dominic J. Hare, Peter J. Crouch, Kay L. Double

**Affiliations:** ^1^ Brain and Mind Centre and Discipline of Pharmacology The University of Sydney, Camperdown Sydney New South Wales 2050 Australia; ^2^ Department of Pharmacology and Therapeutics The University of Melbourne Parkville Victoria 3052 Australia; ^3^ School of BioSciences The University of Melbourne Parkville Victoria 3052 Australia; ^4^ Atomic Medicine Initiative The University of Technology Sydney Broadway New South Wales 2007 Australia

**Keywords:** antioxidants, copper, Cu/Zn superoxide dismutase, neurodegeneration, protein misfolding

## Abstract

Cu/Zn superoxide dismutase (SOD1) is a frontline antioxidant enzyme catalysing superoxide breakdown and is important for most forms of eukaryotic life. The evolution of aerobic respiration by mitochondria increased cellular production of superoxide, resulting in an increased reliance upon SOD1. Consistent with the importance of SOD1 for cellular health, many human diseases of the central nervous system involve perturbations in SOD1 biology. But far from providing a simple demonstration of how disease arises from SOD1 loss‐of‐function, attempts to elucidate pathways by which atypical SOD1 biology leads to neurodegeneration have revealed unexpectedly complex molecular characteristics delineating healthy, functional SOD1 protein from that which likely contributes to central nervous system disease. This review summarises current understanding of SOD1 biology from SOD1 genetics through to protein function and stability.

## Graphical abstract

This review summarizes contemporary understanding of the biology of Cu/Zn superoxide dismutase 1 (SOD1) from SOD1 genetics through to protein function and stability. Our cumulative knowledge of these processes is advancing efforts to identify and target pathways of neurotoxicity driven by atypical SOD1 in multiple incurable neurodegenerative conditions.

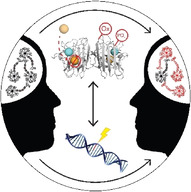

## 1 Introduction

Cu/Zn superoxide dismutase (SOD1) is an intracellular antioxidant enzyme responsible for regulating basal levels of oxidative stress arising from mitochondrial and cytosolic superoxide (O_2_^.−^) production. Its high cytosolic abundance distinguishes it from the other two human superoxide dismutases: Mn superoxide dismutase (SOD2) exclusively localised within mitochondria^[2]^ and extracellular superoxide dismutase (SOD3) that similarly binds Cu and Zn and is anchored to the extracellular matrix.^[3]^ Originally considered to be a Cu storage protein,^[4]^ its crucial role as an intracellular antioxidant was discovered in 1969, when McCord and Fridovich recognised that redox cycling of Cu^+^ bound within the two active sites of the SOD1 homodimer enabled it to effectively convert O_2_^.−^ to O_2_ and H_2_O_2_ by oxidation and reduction, respectively.^[5]^ SOD1 facilitates additional cytoprotective pathways including initiating gene transcription following exposure to neurotoxic stimuli,^[6]^ and physiological roles including modulating signal transduction pathways involving reactive oxygen species (ROS).^[7]^ In contrast SOD1 is also implicated in multiple molecular mechanisms of cytotoxicity, many of which do not simply derive from the abolishment of its cytoprotective functions. Through these mechanisms SOD1 is considered to contribute to pathology in a range of diseases including heart failure,^[8]^ cancer,^[9]^ diabetes,^[10]^ Down's syndrome,^[11]^ amyotrophic lateral sclerosis (ALS)^[12]^ and Parkinson's disease.^[13]^ In particular, the identification of the first *SOD1* gene mutations in a small cohort of familial ALS patients in 1993 signified a major turning point for SOD1 biology, expanding research activities from a simpler understanding of the roles SOD1 plays in normal physiology to focus on the enzyme as a contributory factor to diseases of ageing and neurodegeneration. Advances in our knowledge of SOD1 biochemistry and neurotoxicity are critically appraised in this review. Such advances highlight the potential of treatment strategies aimed at altering or rectifying atypical SOD1 biochemistry, in particular SOD1 metalation, as disease‐modifying therapies for neurodegenerative diseases.

## 2 Genetics and Transcription

**Figure 1:**
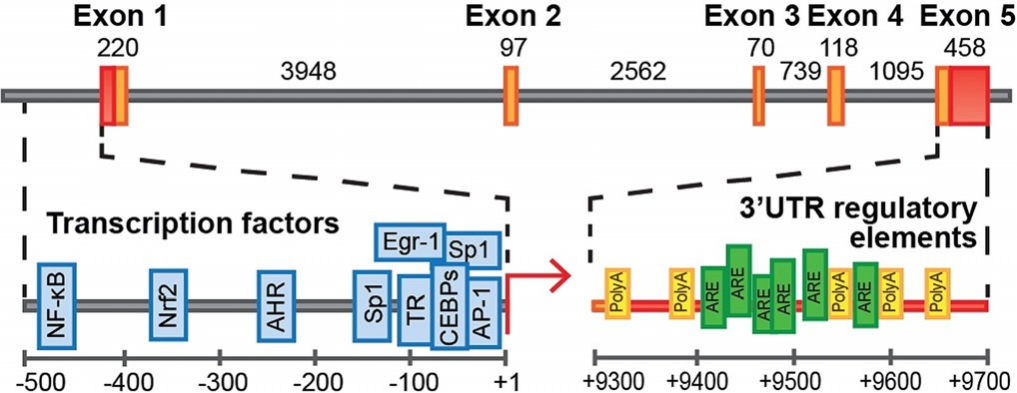
Structure of the human *SOD1* gene and its main regulatory elements. Included: untranslated regions (red), exons (orange), introns (grey), transcription start site (red arrow), transcription factors (blue), polyadenylation (PolyA) sites (yellow) and AU‐rich elements (ARE; green). Exact binding sites for transcription factors are listed in Table 1. Figure adapted from ref. [17]. Copyright Milani et al (2011); Creative Commons Attribution 3.0 licence.

The coding region of the human *SOD1* gene (9307bp, 21q22.11, Entrez Gene ID 6647) consists of five exons interrupted by four introns (Figure 1), which together code for the monomeric SOD1 polypeptide. The 5′ splice donor sequence of the first intron exhibits an unusual variant dinucleotide 5′‐GC compared with the highly conserved 5′‐GT intronic consensus sequence,^[14]^ although this does not affect functionality. The proximal promoter of human *SOD1* mediates basal gene transcription, involving interactions between TATA‐binding protein, CCAAT/Enhancer‐Binding Proteins (C/EBPs)^[15]^ and specificity protein 1,^[16]^ and their corresponding regulatory elements within the proximal promoter: the TATA box, a CCAAT box and a GC‐rich region, respectively (Table 1).^[17]^ Partial overlap between C/EBP and specificity protein 1 binding sequences suggests these transcription factors interact to modulate basal *SOD1* expression, integrating multiple cellular signals into a coordinated response. Moreover, direct interactions of specificity protein 1 with Activating Protein 1 (AP‐1) and Early Growth Response‐1 (EGR‐1) demonstrate additional mechanisms through which SOD1 expression can be regulated.^[18]^

**Table 1:** Transcription factors controlling basal and inducible human SOD1 gene expression.

Transcription factor	*SOD1* recognition sequence	Biochemical cue(s)	Regulatory impact
TATA‐binding protein (TBP)	TATA box	NA	constitutive expression^[16]^
CCAAT/Enhancer Binding Proteins (C/EBPs)	CCAAT box (−64 bp to −55 bp)	NA	constitutive expression^[14]^
Specificity Protein 1 (Sp1)	GC‐rich domains (−104 bp to −89 bp and −59 bp to −48 bp)	NA	constitutive expression^[15]^
Early Growth Response‐1 (Erg1)	GC‐rich domain (−59 bp to −48 bp)	O_2_^.−^ production via phorbol‐12‐myristate‐13‐acetate administration^[19]^	positive
Aryl Hydrocarbon Receptor (AHR)	Xenobiotic response element (−255 bp to −238 bp)	Synthetic halogenated and nonhalogenated aromatic hydrocarbons (e.g. TCDD)^[20]^	positive
Nuclear Factor E2‐Related Factor 2 (Nrf2)	Antioxidant responsive element (−356 bp to −330 bp)	Reactive oxygen species (O_2_^.‐^), low‐dose non‐toxic proteasome inhibition^[21]^	positive
Nuclear Factor‐KappaB (NF‐κB)	p65‐NF‐κB binding site (−552 bp to −355 bp)	Exposure to cytokines, radiation, oxidative stress (H_2_O_2_)^[22]^	positive
Activating Protein 1 (AP1)	NA—reduces Sp1 binding^[23]^	Increased neuronal nitric oxide synthase, growth factors, cytokines, oxidative stress	negative
Thyroid Hormone Receptor (TR)	Thyroid hormone inhibitory element (−157 bp to +17 bp)	Thyroid hormones bind to receptor, complex inhibits SOD1 transcription, unliganded receptor induces transcription^[24]^	negative

NA=not available.

Two SOD1 mRNAs (0.7kb and 0.9kb) differing in the length of their 3′ untranslated regions (UTRs) have been identified in human cells,^[17]^ and are a product of two distinct groups of mRNA processing/polyadenylation signals within the 3′UTR (Figure 1). Located within 0–76bp and 200–250bp of the stop codon, the first group mediates the production of the 0.7kb mRNA, whilst the second produces the 0.9kb mRNA.^[14]^ Both mRNA species are functional, as they can be translated in vitro to produce SOD1 protein; however, the shorter transcript is four times more abundant than its longer counterpart.^[14]^

Primer extension and S1 nuclease mapping demonstrate similar variation in the 5′ terminus of SOD1 mRNA.^[19]^ SOD1 mRNA primarily possesses a 5′ transcription start site 23bp downstream of the proximal promoter TATA box (TATAAA), reinforcing the importance of TATA‐dependent transcription for *SOD1* gene expression. A range of other less abundant SOD1 mRNA molecules have also been identified with 5′ termini 30, 50 and 65bp upstream of the major transcription site, indicating TATA‐independent *SOD1* expression. The specific cellular conditions which dictate the mechanism of *SOD1* transcription, as well as the functional importance of the variable 5′ and 3′ termini, are currently unknown. This variability may result in subtle functional differences between SOD1 mRNAs, which are likely products of the different functional requirements of distinct cell types (neurons vs. glia), or tissue regions (brain vs. liver).^[17]^

In addition to control of its constitutive expression, induction of the human *SOD1* gene is modulated by intracellular molecular events (redox sensing). Biochemical cues are transduced into a transcriptional response by *trans*‐acting transcription factors and known *cis*‐regulatory DNA elements within, and surrounding, the human *SOD1* gene, identified through genetic structure/function analyses (Figure 1, Table 1).

## 3 Post‐transcriptional Regulation

Augmenting regulatory control of *SOD1* transcription, post‐transcriptional factors such as mRNA processing, nuclear export, mRNA stability, translational efficiency and microRNA (miRNA)‐dependent modulation are likely to influence the abundance of specific SOD1 mRNA transcripts within a cell. Most mRNA regulatory elements generally exist in the 5′ and 3′UTRs, where interactions within the 5′UTR primarily regulate mRNA translation whilst those within the 3′UTR modulate mRNA stability and metabolism.^[20]^ With respect to SOD1 mRNA, little is known regarding the presence or influence of many of these post‐transcriptional regulatory mechanisms. The two distinct human SOD1 mRNA transcripts previously discussed are suggested to arise from 3′UTR A/U‐rich elements (AREs), indicating several known ARE‐binding proteins may function as *trans*‐acting regulatory factors influencing SOD1 mRNA stability and translation.^[21]^ Ascertaining the presence and identity of these factors will be important to advance our understanding of SOD1 mRNA regulation. MiRNA‐mediated regulatory control may also play a key role in SOD1 mRNA regulation at the 3′UTR, with computational and biological approaches recently identifying SOD1 as a target of miR‐377 and miR‐206, which inhibit SOD1 protein production.^[22]^ The relevance of miRNAs for physiological *SOD1* expression remains an area of SOD1 biology that requires further exploration. Our understanding of the functional relevance of variability in the 5′UTR of SOD1 mRNA is similarly lacking; however, 5′UTR variability appears to govern mRNA capping to regulate nuclear export, digestion by exonucleases, and ribosomal binding. It remains to be seen whether this is the case with SOD1 mRNA.

## 4 Protein Structure and Exceptional Stability

**Figure 2:**
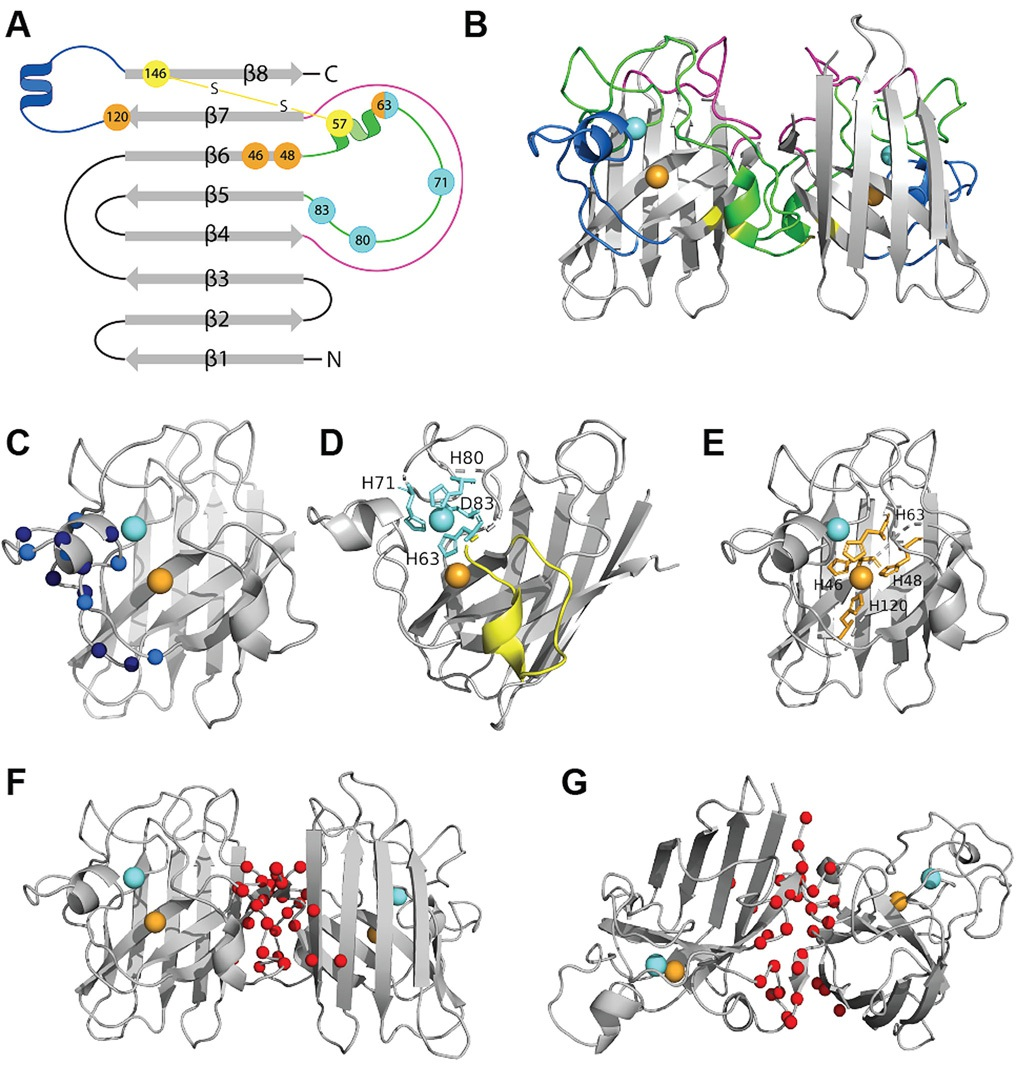
Structural elements within SOD1 protein. A,B) Mature SOD1 is dimeric, and comprises an eight‐stranded β‐barrel (grey) with one Cu (orange) and Zn (cyan) ion bound in each monomer. The electrostatic loop (blue; A, B, C) contains charged (dark blue) and polar (light blue) residues important for guiding anionic O_2_^.−^ towards the active site. The metal‐binding loop (green; B) mediates Zn coordination through three histidine residues and one aspartic acid residue (cyan; A, D). The disulfide loop (yellow; D) is a substructure within the metal‐binding loop, and contains one of the two cysteine residues forming a stabilizing intramonomeric disulfide bond (yellow; A, B). Cu coordination is facilitated by four histidine residues (orange; A, E), one of which is shared with Zn (His63). Multiple residues within β‐strand 8 and the disulfide loop participate in reciprocal hydrogen bonding between SOD1 monomers (red; F, G), creating a tight interface that underlies the exceptional thermodynamic stability of the holo‐SOD1 dimer. The Greek key loop (pink; A, B) forms a plug at one pole of the β‐barrel and contributes to dimer interface stability. Exact residues pertaining to each structure detailed in Supplementary Table 1.

Mature, functional human SOD1 is a relatively small (32 kDa) homodimeric metalloprotein. Following translation, monomeric SOD1 (153 amino acids, 16 kDa, UniProtKB P00441), folds into an eight‐stranded Greek‐key β‐barrel (Figure 2; Supplementary Table 1).^[23]^ Strands 1, 2, 3 and 6 are on the opposite side of the β‐barrel to the active site, and are regular with minimal torsion. In contrast, strands 4, 5, 7 and 8 are shorter and exhibit greater torsion, and contain β bulges which accommodate metal binding to form the active site. Two large loops, the electrostatic loop (residues 122–143) and the metal‐binding loop (residues 49–84), play important functional roles in SOD1 catalysis and protein folding, respectively (Figure 2 A–D). Charged (Lys, Arg, Asp, Glu) and polar (Asn, Ser, Thr) residues within the electrostatic loop (loop VII; Figure 2 C) create a positive electric field in the channel leading towards the active site in mature SOD1 protein, enhancing enzyme function by providing electrostatic guidance to anionic O_2_^.−^ towards solvent‐accessible catalytic Cu^2+^ in the active site.^[24]^ Mutagenic studies demonstrate that the specific folding and orientation of the electrostatic loop, as well as the location and identity of charged residues within it, strongly influence its ability to augment catalytic activity.^[25]^ The metal‐binding loop (loop IV; Figure 2 D) contains histidine (His63, His71, His80) and aspartic acid (Asp83) residues that mediate Zn coordination in a distorted tetrahedral arrangement, which contributes to the formation of the active site during SOD1 maturation (Figure 2). Residues 49–62 within the metal‐binding loop form a substructure termed the disulfide loop, containing Cys57 which, together with Cys146 from β‐strand 8, form a conserved disulfide bond within each SOD1 monomer.^[26]^ This stable disulfide is incredibly rare within the reducing environment of the cytosol where SOD1 resides, and is the only known example of this bond to exist in an oxidised state in a mature, functional protein in this compartment. Coordination of the catalytic co‐factor, Cu, within the active site is mediated by histidine residues (His46, His48, His63, His120) primarily located in the core of the protein (Figure 2 E), as well as a fifth water ligand that completes a square‐pyramidal coordination arrangement. The utilisation of four histidine residues in the Cu binding site is required for both the binding of Cu^+^ and the facilitation of Cu redox cycling involved in SOD1 activity.^[27]^ Finally, a third and shorter loop, known as the Greek key loop (residues 102–115), forms a plug at one pole of the β‐barrel and contributes to dimer interface stability.

Human SOD1 protein exists within neurons as a mixture of monomeric and dimeric metal‐free (apo‐), partially metalated and fully metalated (holo‐)SOD1 metalloforms, with monomeric apo‐SOD1 constituting the most immature SOD1 metalloform. SOD1 maturation involves the dimerisation of two apo‐SOD1 monomers to form a holo‐SOD1 dimer, which is dependent upon key post‐translational modifications to the immature apo metalloform: the binding of one Zn and Cu ion per monomer, intramonomeric disulfide bridging and homodimerisation. Protein dimerisation buries approximately 640 Å^2^ of hydrophobic surface area at the dimer interface through a complex network of hydrogen bonds and hydrophobic interactions,[^23^, ^26^] creating a compact mature homodimer with minimal solvent accessibility. Accordingly, holo‐SOD1 is one of the most stable proteins known,^[28]^ with a high buffer‐dependent melting point of 85–95 °C^[29]^ and an enzymatic activity that is retained in chemical environments as harsh as 8 M urea or 4 M guanidine‐HCl.^[30]^ Interestingly, the melting temperature of apo‐SOD1 (*T_m_*=42.9 °C) is only slightly higher than physiological temperature (≈37 °C),^[31]^ suggesting that the metal‐free protein exists on the cusp of thermodynamic unfolding under physiological conditions. The introduction of *SOD1* mutations, or alteration of side‐chain post‐translational modifications (e.g. cystine/histidine oxidation) over time, as discussed in subsequent sections of this review, can reduce the *T_m_* of apo‐SOD1 below this threshold to trigger complete protein unfolding at normal body temperature. It is perhaps for this reason that the less stable apo‐SOD1 is more rapidly degraded under physiological conditions by both the 20S proteasome and macro‐autophagy than its holo‐SOD1 contemporary.^[32]^

The presence of bound Zn and Cu, and of a fully formed disulfide bond, is of critical importance to the remarkable stability of dimeric holo‐SOD1. Stabilisation of the dimer interface originates primarily from reciprocal connections between disulfide loop residues 50–54, Greek key loop residues (residues 102–115) and residues within the C‐terminal β‐strand of each SOD1 monomer (residues 148, 150–153; Figure 2 F,G).^[26]^ Ile151 residues, in particular, form strong hydrogen bonds with Gly51 and Gly114 from opposing monomers, creating four hydrogen bonds across the interface. NMR spectroscopy,^[33]^ crystallography^[34]^ and hydrogen/deuterium exchange studies^[35]^ report substantial disorder of these structural elements, as well as of the electrostatic loop,[^33c^, ^33d^, ^34^] within apo‐SOD1 in the absence of bound metals and an oxidised disulfide bond. Metal binding and disulfide bond formation counteract this instability by facilitating the formation of stabilizing hydrogen‐bonding networks, comprehensively described by Tainer and colleagues,[^23^, ^36^] which reduce structural disorder by anchoring key structural motifs to primary or secondary metal or disulfide ligands.

Residues that hydrogen bond to Zn ligands are found within the metal‐binding (Asn65, Arg69, Lys70, Arg79, His80, Asp83) and electrostatic (Asp124, Thr135) loops,[^23^, ^36^] and solution NMR spectroscopy demonstrates that zinc binding elicits structural rigidity of these motifs comparable to that found in mature holo‐SOD1.^[33c]^ These structural changes, in combination with Zn binding to His63 at the edge of the disulfide loop, significantly promote disulfide bond formation between disulfide loop Cys57 and Cys146 at the base of the electrostatic loop.^[37]^ The formation of this bond anchors the disulfide and zinc loops to the core of SOD1 protein, and promotes SOD1 homodimerisation through the formation of strong hydrogen bonds between disulfide loop Gly51 residues and Ile151 residues in the C‐terminal β‐strands of pairing monomers.^[38]^ The thermodynamic stabilizing effect of Zn^2+^ on apo‐SOD1 (Δ*T_m_*=+15.5 °C, *T_m_*=58.4 °C) is greater than that afforded by disulfide formation alone (Δ*T_m_*=+6.9 °C, *T_m_*=49.8 °C), with their combined influence larger again (Δ*T_m_*=+31.7 °C, *T_m_*=74.6 °C).^[31]^ The greatest increase in stability, however, occurs following the combination of Zn^2+^ binding and disulfide bond formation with Cu^+^ binding and homodimerisation (Δ*T_m_*=+42.1–52.1 °C, *T_m_*=85–95 °C, buffer‐dependent).

Zn binding and disulfide status are traditionally considered the key determinants of SOD1 stability; however, Cu plays a dominant role in the kinetic stability of SOD1 protein.^[39]^ This may be attributed to the larger number of structural elements directly involved in hydrogen‐bonding networks with Cu ligands compared with Zn ligands. Cu^+^ binding improves monomer (and therefore dimer) stability by initating hydrogen bonding which limits 1) β‐barrel and C‐terminal β‐strand mobility within each monomer (His43, Gly44, Thr116, Val118); 2) disulfide loop mobility by anchoring it to the β‐barrel (His48‐Gly61); 3) electrostatic loop mobility by anchoring it to the disulfide loop (Cys57‐Arg143‐Gly61) and β‐barrel (His120‐Gly141).[^23^, ^26^, ^36^] Cu‐induced conformational changes therefore augment structural stabilisation of the metal‐binding and electrostatic loops elicited by Zn binding and, together with disulfide bond formation, complete hydrogen‐bonding networks across the dimer interface.

## 5 Post‐translational Processing

The maturation pathway of monomeric apo‐SOD1 to a functional holo‐SOD1 dimer is complex and incompletely described. Over 44 conformations of the SOD1 polypeptide are theoretically possible,^[31]^ depending upon metal occupancy, disulfide status, and oligomeric state. Not all conformations are energetically favourable and the existence of most conformers remains purely theoretical under physiological conditions. SOD1 maturation therefore remains best understood in terms of transitional free‐energy states. Sophisticated NMR techniques enable the construction of structural, kinetic and thermodynamic profiles of apo, holo and partially metalated SOD1 protein metalloforms. Free‐energy landscapes of each metalloform can then be constructed which depict all interrelated transition‐state conformations for a given metalloform, providing indications of energetically favourable routes of protein maturation.

**Figure 3:**
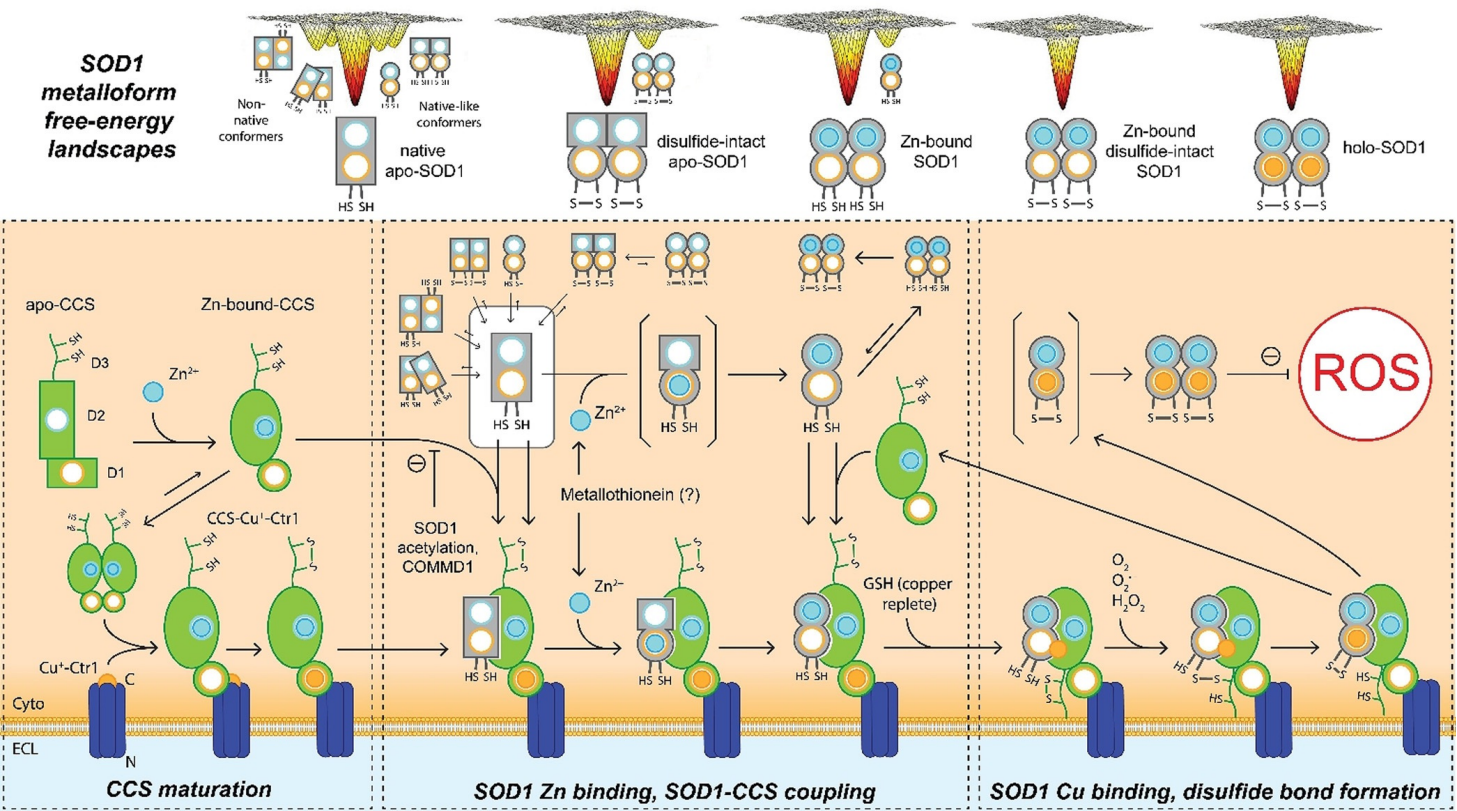
Transitional free‐energy landscapes of SOD1 metalloforms and CCS‐dependent SOD1 maturation. Free energy landscapes of SOD1 metalloforms demonstrate the existence of multiple conformers, which interchange in complex equilibria. Zn incorporation into the native monomeric SOD1 and CCS apo proteins occurs via unknown mechanisms, and elicits conformational change in each protein to prepare them for coupling and Cu^+^ insertion. Zn insertion into apo‐CCS prompts homodimerisation and migration to the plasma membrane, where it forms a stable complex with the copper transporter Ctr1 via its C‐terminal domain (CCS‐Cu^+^‐Ctr1) and acquires Cu^+^ in D1. Zn incorporation into apo‐SOD1 is promoted by CCS coupling, mediated by D2 and D3 of CCS protein. Apo‐ or Zn‐bound‐SOD1 may couple with CCS within a preformed CCS‐Cu^+^‐Ctr1 complex at the plasma membrane, forming a ternary SOD1‐CCS‐Cu^+^‐Ctr1 complex, or alternatively may couple with CCS within the cytosol and subsequently migrate to the plasma membrane to complex with Ctr1. Zn first binds transiently to the Cu‐binding site of SOD1, triggering the formation of the Zn‐binding site and the subsequent migration of Zn to its physiological Zn centre. Zn binding reduces structural flexibility in SOD1 protein and prevents transitions to non‐native excited SOD1 conformers. SOD1‐CCS coupling and SOD1 Zn binding trigger conformational change within CCS, whereby D3 arches over the disulfide loop of SOD1 to stabilise SOD1‐CCS coupling, uncover an “entry” binding site for Cu^+^ in SOD1, and expose the SOD1 Cys146 residue to solvent. Oxidation of the exposed thiol side chain of SOD1 Cys146 triggers disulfide exchange reactions between the CXC motif in CCS D3, and adjacent Cys146/Cys57 residues of SOD1 protein, culminating in the formation of a disulfide bond between SOD1 Cys57 and Cys146. SOD1 disulfide bond formation promotes Cu^+^ migration from the entry site to the active site. Together these events trigger SOD1 and CCS dissociation from Ctr1, producing fully metalated holo‐SOD1 monomers that can now homodimerize.

Disulfide‐reduced apo‐SOD1 exists in equilibrium with four different conformers; two resemble the native dimer, whilst the other two are described as non‐native oligomers predicted to have a propensity towards aberrant interactions, including aggregation (Figure 3).[^33c^, ^33d^] These species are clearly of sparse abundance,[^33c^, ^33d^] whilst the native β‐barrel monomeric state of disulfide‐reduced apo‐SOD1 is the most abundant form. CD and NMR spectroscopy reveal that the β‐barrel folding pattern of the dominant conformer is acquired prior to post‐translational modifications, and that this conformer possesses a low α‐helical content with substantial disorder of the metal binding and electrostatic loops.^[40]^ Zn insertion into the apo‐SOD1 monomer initiates protein maturation, quenching the two non‐native conformers following stabilisation of the electrostatic and metal binding loops (Figure 3). Zn‐bound SOD1 exists in equilibrium between monomeric and dimeric conformers, with the dimeric conformer strongly favoured over the monomer at physiological concentrations.^[40]^ Sudden increases in oxidative stress rapidly induce SOD1 antioxidant activity,^[41]^ suggesting rising ROS levels trigger Zn insertion into the labile apo‐SOD1 pool to mitigate ROS‐induced damage. To date, the molecular mechanism of Zn insertion is not well understood. Given intraneuronal Zn concentrations are controlled with femtomolar sensitivity,^[42]^ it is likely that Zn is chaperoned to SOD1 rather than acquiring it from a labile diffusible pool. Recent in vitro and in‐cell NMR spectroscopy data demonstrate that zinc acquisition by apo‐SOD1 is promoted by an interaction with copper chaperone for SOD1 (CCS) protein (Figure 3).^[43]^ It is unclear whether Zn is transferred to apo‐SOD1 from CCS, or whether structural change in apo‐SOD1 induced by this interaction promotes high‐affinity zinc binding from a third party, such as metallothionein.^[44]^

Following Zn^2+^ binding, Cu^+^ is inserted into the active site of the SOD1 monomer, primarily through a highly specific and complex interaction with CCS protein (Figure 3), which is closely tied to the formation of the stabilising intramolecular disulfide bond in SOD1 protein. In this way, CCS constitutes an essential molecular chaperone, facilitating multiple stages of SOD1 maturation. Together, metalation and disulfide bond formation eliminate all four excited states available to the apo‐SOD1 monomer, producing a smoothened free energy landscape for the fully mature holo‐SOD1 dimer exhibiting minimal opportunity for aberrant folding or interactions.

### 5.1 CCS‐Dependent SOD1 Maturation

**Figure 4:**
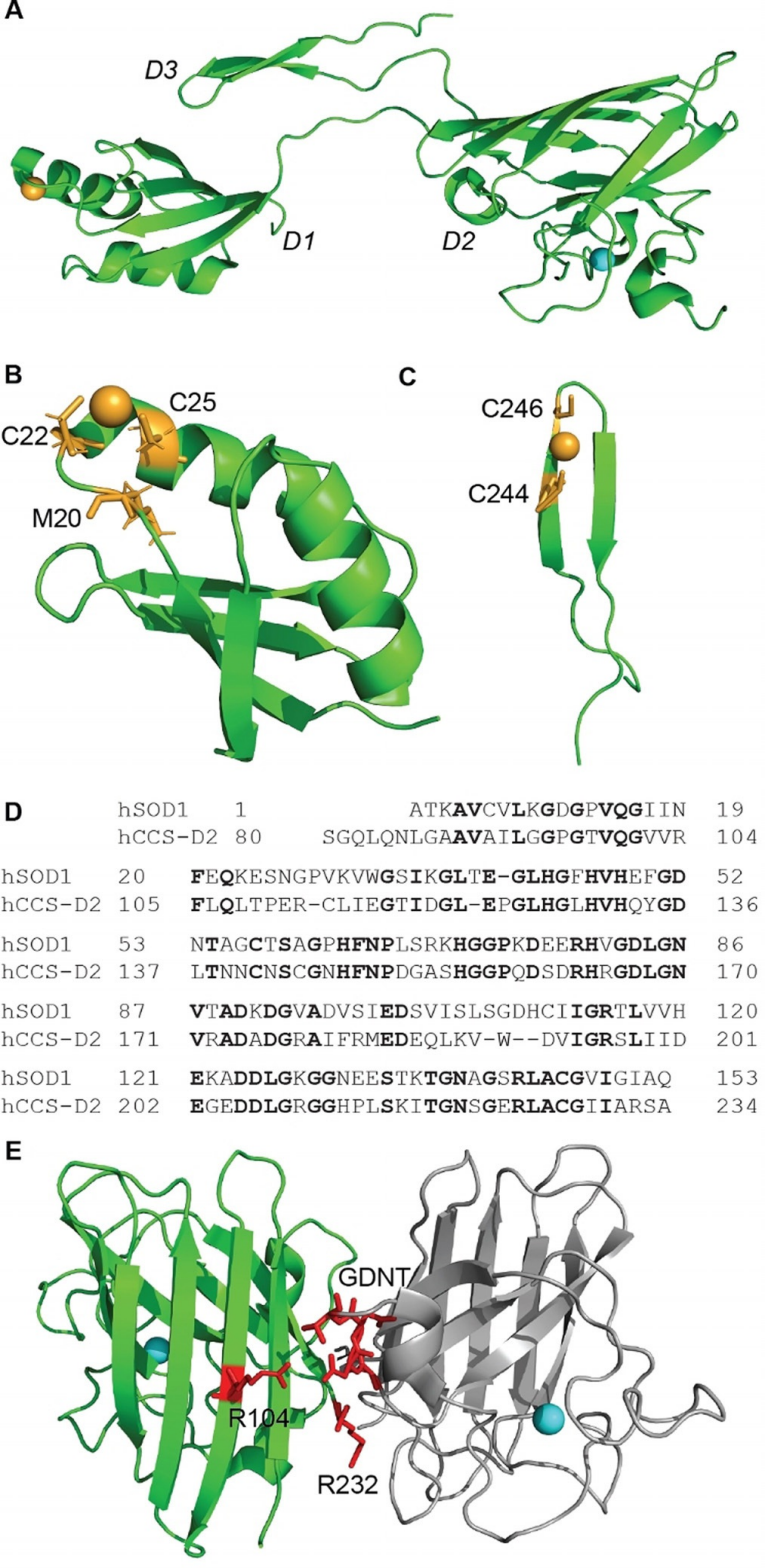
Structural elements within CCS protein and its structural homology to SOD1 protein. A) CCS protein consists of three distinct domains (D1–D3). D1 (B) and D3 (C) bind Cu^+^ (orange spheres) by virtue of MXCXXC and CXC binding motifs, respectively. D) D2 exhibits a high sequence homology to SOD1 protein (homologous residues indicated by black bolding), facilitating SOD1–CCS heterodimerisation prior to Cu transfer and disulfide bond formation. E) Conserved arginine residues at positions 104 and 232 of CCS D2, together with a GDNT motif within SOD1 protein, mediate strong stabilizing interactions across the heterodimer interface (residues highlighted in red). Zn^2+^ represented by cyan spheres in (A) and (E).

Human CCS is a larger homodimeric metalloprotein compared with SOD1 that is widely expressed in eukaryotes,^[45]^ albeit at much lower levels than SOD1. Each CCS monomer (273 amino acids, 29 kDa, UniProtKB O14618) binds one Cu^+^ and Zn^2+^ and consists of three distinct domains (D1–D3; Figure 4 A) that mediate key aspects of the SOD1–CCS interaction. Prior to acquiring Cu, CCS binds one Zn^2+^ ion in D2 of each monomer,^[46]^ eliciting structural change in this domain to presumably prepare the protein for Cu incorporation. Similar to SOD1, the trigger and mechanism for Zn acquisition by CCS are not well understood. Homodimerisation of Zn‐bound CCS promotes its localisation to the plasma membrane,^[47]^ where Cu^+^ acquisition may occur directly from the C‐terminus of high‐affinity Cu uptake protein 1 (Ctr1),^[48]^ or via an intermediary chaperone such as reduced glutathione or Atox1 when cellular Cu is more abundant (Figure 3).^[49]^ CCS contains two conserved Cu‐binding motifs in D1 (MXCXXC; Figure 4 B) and the D3 β‐hairpin loop (CXC; Figure 4 C) of each monomer.^[50]^ The strong Cu binding affinity of cysteine enables high‐affinity Cu^+^ binding by CCS using fewer residues than the histidine‐rich SOD1 Cu binding site (Figure 2 E).^[27]^ D1 is likely to dominate Cu sequestration by CCS, as well as Cu^+^ transfer to SOD1, owing to its superior Cu^+^ affinity compared with CCS D3, yet lower Cu^+^ affinity compared with SOD1.^[50]^

Recent data suggests that CCS‐mediated metalation and activation of SOD1 likely occurs on the cytosolic side of the plasma membrane following CCS Cu^+^ acquisition from Ctr1,^[48]^ involving a ternary SOD1‐CCS‐(Cu^+^)‐Ctr1 complex (Figure 3). Complex formation in vitro requires a direct interaction between CCS‐(Cu^+^)‐Ctr1 and either apo‐ or Zn‐bound‐SOD1, or alternatively may occur from an interaction between preformed cytosolic CCS‐Zn‐bound‐SOD1 heterodimers and Cu^+^‐Ctr1. The lack of a direct interaction between Cu^+^‐Ctr1 and Zn‐bound‐SOD1^[48]^ reinforces the importance of CCS as a molecular chaperone for immature SOD1 metalloforms. It is difficult to determine which of the two complexation mechanisms is more physiologically relevant in vivo. In‐cell NMR spectroscopy reveals that CCS exhibits a much higher binding affinity for apo‐SOD1 compared with Zn‐bound SOD1 in human cells,^[43b]^ indicating that apo‐SOD1 likely either couples with CCS in the cytosol prior to zinc acquisition and complexation with Cu^+^‐Ctr1, or that it couples with the CCS‐(Cu^+^)‐Ctr1 complex at the plasma membrane. The binding affinity of the CCS‐(Cu^+^)‐Ctr1 complex for apo‐SOD1 is equivalent to that of CCS alone.^[48]^ Importantly, crystallographic data demonstrate that Zn‐bound‐SOD1 monomers and CCS‐Zn‐bound‐SOD1 heterodimers have a much lower affinity for lipid bilayers compared with CCS homodimers or apo‐SOD1 alone,^[47]^ a factor not taken into account in recent studies of the ternary SOD1‐CCS‐(Cu^+^)‐Ctr1 complex.^[48]^ Considering these data, the most probable explanation is that apo‐SOD1 directly interacts with the CCS‐(Cu^+^)‐Ctr1 complex at the plasma membrane, where it acquires both Zn and Cu following a direct interaction with CCS. It is possible that preformed CCS‐apo‐SOD1 heterodimers may also complex with Cu^+^‐Ctr1, similar to Zn‐loaded heterodimers; however, this has not been investigated.

Of the three domains of human CCS, D2 is most important in SOD1 protein recognition, exhibiting a high sequence homology to human SOD1 (Figure 4 D).^[50a]^ Evolutionarily conserved arginine residues at positions 104 and 232 within D2 of human CCS, together with a Gly51‐Asp52‐Asn53‐Thr54 (GDNT) motif within the disulfide loop of human SOD1, mediate stabilizing hydrogen‐bonding interactions across the heterodimer interface (Figure 4 E).^[47]^ Important roles for D1 and the D3 β‐hairpin loop of CCS protein in SOD1–CCS recognition have also more recently been identified in structural and spectroscopic studies utilising the yeast SOD1 and CCS homologues Sod1 and Ccs1. D1 of Ccs1 protein is thought to stabilise the flexible D3 β‐hairpin loop by anchoring the D3 CXC motif (Figure 4 C) in a narrow pocket between itself, and both the disulfide loop and β‐barrel of Sod1.[^43c^, ^51^] This effectively expands and strengthens the Sod1–Ccs1 interface to yield a 7–15‐fold improvement in Sod1–Ccs1 binding affinity,^[43b]^ and positions the CCS D3 cysteine residues adjacent to the SOD1 disulfide loop Cys57 residue in preparation for disulfide bond formation. These data may underlie the higher binding affinity of CCS for apo‐SOD1 compared with Zn‐bound‐SOD1 (Figure 3),^[43b]^ given greater structural flexibility of the disulfide loop in apo‐SOD1 likely promotes CCS D3 intercalation to strengthen the SOD1–CCS interface. Such a mechanism may also clarify how CCS promotes and/or facilitates high‐affinity zinc binding to apo‐SOD1 in human cells (Figure 3),[^43a^, ^43b^, ^52^] whereby structural changes in CCS D3 are proposed to stabilise the metal‐binding loop of SOD1 protein to generate a complete SOD1 Zn‐binding site ready for Zn^2+^ incorporation.^[43c]^

Intriguingly, in a separate experimental program, Leinartaite and colleagues demonstrated that human apo‐SOD1 folding in a bacterial expression system co‐expressing Ccs1 is initiated by transient coordination of Zn^2+^ to the Cu ligands of apo‐SOD1.^[53]^ This triggers global structural rearrangement of the apo protein, culminating in the formation of the higher affinity Zn site approximately 6.3 Å away, to which Zn eventually migrates (Figure 3). Given structural changes in apo‐SOD1 protein elicited by CCS binding promote Zn^2+^ incorporation into apo‐SOD1,[^43a^, ^43b^] it is possible that CCS binding to apo‐SOD1 may facilitate the initial binding of Zn^2+^ to the Cu ligands of apo‐SOD1. Alternatively this mechanism may promote structural rearrangement of apo‐SOD1, enabling eventual migration of Zn^2+^ to the higher affinity Zn site. Again it is unclear whether CCS or a third‐party chaperone constitute the Zn source. Clarifying possible relationships between these recent structural advances may advance our understanding of the mechanism of Zn incorporation into apo‐SOD1.

Structural changes elicited by Zn binding alter the conformation of the active site and disulfide loop, and thus Zn binding must occur before Cu^+^ binding and disulfide bond formation can ensue.^[50a]^ Similar to Zn binding, CCS plays a primary role in both SOD1 Cu^+^ binding and disulfide bond formation under physiological conditions.^[54]^ Mirroring other aspects of Zn coordination, recent crystallographic and spectroscopic data obtained by Fetherolf and colleagues demonstrate that Cu^+^ may be initially bound by SOD1 at an “entry site”, and that subsequent migration of Cu^+^ to the higher affinity active site is triggered by structural changes linked with disulfide bond formation.^[51]^ Formation of the entry site is proposed to follow the intercalation of the D3 β‐hairpin loop between the disulfide loop and β‐barrel of SOD1 protein during CCS–SOD1 coupling (Figure 3). Together with hydrogen‐bonding interactions between the side chain of SOD1 disulfide loop residue Asp52 and the indole nitrogen of aromatic residues in CCS D3, β‐hairpin intercalation pulls the disulfide loop away from the β‐barrel of Sod1, exposing the Cu^+^‐binding entry site and an adjacent electropositive cavity harbouring the Cys146 residue of SOD1 protein.^[51]^ Cu^+^ coming from CCS D1 or an alternative cuprochaperone, such as glutathione,[^49a^, ^55^] can now bind at the entry site, and either O_2_^.−^ or H_2_O_2_ can diffuse toward the electropositive cavity harboring SOD1 Cys146. H_2_O_2_ within the electropositive cavity, whether it originates from direct diffusion of H_2_O_2_ into the cavity or from Cu^+^‐induced breakdown of O_2_^.−^ into H_2_O_2_ and water within the cavity, is proposed to promote sulfenylation of the SOD1 Cys146 thiol side chain within the cavity, which subsequently triggers SOD1 disulfide bond formation (Cys57–Cys146) through disulfide exchange reactions with CCS D3 cysteines positioned adjacent to the cavity.^[51]^ It is likely that either Cys146 sulfenylation, disulfide bond formation, or a combination of both events, drive Cu^+^ migration from the entry site to the tetrahistidine environment of the active site. SOD1 disulfide bond formation and Cu^+^ binding at the active site expel the stabilising CCS D3 β‐hairpin from the SOD1–CCS interface^[51]^ and disrupt hydrogen‐bonding interactions between CCS D2 Arg232 and the SOD1 GDNT motif.^[47]^ This closes the electropositive cavity and triggers dissociation of SOD1 and CCS from their ternary complex with Ctr1 at the plasma membrane,^[48]^ producing fully metalated holo‐SOD1 monomers that can now homodimerize. This proposed mechanism has yet to be independently verified but is consistent with the vast majority of biochemical data on CCS‐mediated SOD1 activation in the literature.[^43b^, ^43c^, ^47^, ^48^, ^50a^, ^56^]

Importantly, neither sulfenylation^[51]^ nor disulfide bond formation^[57]^ in human SOD1 are entirely dependent on CCS, and this will be discussed in subsequent sections of this review. Atomic resolution monitoring of CCS‐dependent SOD1 maturation using in‐cell NMR spectroscopy further demonstrates that CCS‐dependent SOD1 disulfide formation may also occur in the absence of copper, albeit to a lesser extent (≈50 %) than under copper‐replete conditions.^[56]^ This is curious given that crystallographic data indicates SOD1–CCS coupling prevents premature disulfide bond formation in Zn‐bound SOD1.^[47]^ CCS coupling disrupts existing hydrogen bonds between SOD1 disulfide loop Gly61 and Cys57 carbonyls and SOD1′s Arg143 guanidinium group, resulting in Arg143 migration and hydrogen bonding to GDNT tetrad Asn53. Consequently, Arg143 interposes between disulfide bond residues Cys57 and Cys146 and greatly increases solvent accessibility of the SOD1 Cu‐binding site,^[47]^ ensuring the amenability of the active site to receive Cu whilst simultaneously preventing premature disulfide bond formation. Again, investigating possible relationships between these structural features may improve our understanding of mechanisms of Cu^+^ incorporation and disulfide bond formation during SOD1 maturation.

### 5.2 CCS‐Independent SOD1 Maturation

Following its discovery in 1997, CCS was believed to be the sole means of SOD1 activation in vivo.^[58]^ Three years later, mouse strains bearing a homozygous CCS deletion were reported to exhibit a small but persistent degree of SOD1 activity,^[54]^ indicating CCS‐independent mechanisms of Cu delivery to SOD1. Recently, an elegant study in yeast found no apparent differences in the kinetics of Cu activation in the presence and absence of CCS, and determined that CCS‐dependent and ‐independent pathways likely obtain Cu from the same upstream source.^[57]^ CCS‐independent mechanisms were also shown to mediate partial disulfide bond formation (40–50 %) in inactive human SOD1 during conditions of Cu starvation and/or CCS deficiency, demonstrating that disulfide bond formation is not solely reliant on CCS‐mediated Cu incorporation. This pathway of CCS‐independent SOD1 activation was even observed under hypoxic and anoxic conditions, illustrating that SOD1 activity can be maintained over a range of oxygen conditions. The authors attribute the presence of these two distinct but convergent pathways to the dual role of SOD1 in oxidative stress protection and neuronal signaling.^[59]^ Oxygen‐regulated Cu incorporation by CCS is proposed to be necessary under aerobic conditions when oxidative stress is a concern, and maximal SOD1 activity is required.^[57]^ In contrast, CCS‐independent SOD1 activation may be prioritised during conditions of low oxygen and low oxidative stress, where the reactants and products of SOD1 catalysis have a greater influence on neuronal signaling processes, which will be discussed in subsequent sections of this review. In the latter scenario, SOD1 activity is diminished through a gradual tapering of CCS‐dependent activation, with minimal SOD1 activation facilitated through CCS‐independent pathways. Specific down‐regulation of CCS‐dependent SOD1 activation and activity may be mediated by a novel interaction with COMMD1 protein, shown to interact with SOD1 protein to reduce homodimer formation and diminish antioxidant activity in vitro.^[60]^ *SOD1* gene mutations enhance this interaction, implicating COMMD1 in the accumulation of misfolded SOD1 monomers which are likely to drive neurotoxic SOD1 aggregation;^[61]^ this will be discussed below in subsequent sections of this review. Further, acetylation of SOD1 at Lys70 impairs SOD1–CCS recognition and diminishes its enzymatic activity, changing the net charge of Lys70 within the electrostatic loop from approximately +1 to 0. Lys70 may be deacetylated by SIRT1, suggesting a role in regulating CCS‐dependent SOD1 function,^[62]^ especially within the nucleus where SIRT1′s deacetylase activity is more prominent.

Reduced glutathione (GSH) and protein deglycase DJ‐1 may constitute two alternative sources of SOD1 Cu loading and activation. GSH is implicated in the delivery of Cu to SOD1 in CCS^−/−^ mice,^[55]^ and has been suggested to acquire Cu directly from Ctr1, acting as an intermediary chaperone between Ctr1 and cuproproteins (SOD1) or primary cuprochaperones (CCS, metallothionein, Atox1).^[49a]^ Measurement of the abundance and Cu binding affinities of GSH, various Cu chaperones and their target proteins in vitro support a model in which cytosolic GSH receives Cu and transfers it to higher affinity binding sites on less abundant cuprochaperones and proteins according to a positive affinity gradient.[^49a^, ^63^] Cu‐loaded wild‐type DJ‐1 elicits a three‐fold elevation in SOD1 activity upon incubation with Zn‐bound apo‐SOD1 in vitro,^[64]^ implying substantial activation of SOD1 via Cu insertion. A direct interaction has also been reported between DJ‐1 and SOD1 using pull‐down assays.[^64^, ^65^] In contrast, DJ‐1 did not bind copper, nor activate SOD1, in cultured human cells in a more recent investigation using in‐cell NMR spectroscopy.^[66]^ The specific mechanisms of GSH and DJ‐1 in SOD1 activation remain ongoing areas of investigation,^[67]^ and may represent important mediators of CCS‐independent SOD1 metalation and disulfide bond formation.

## 6 SOD1 Protein Function

**Figure 5:**
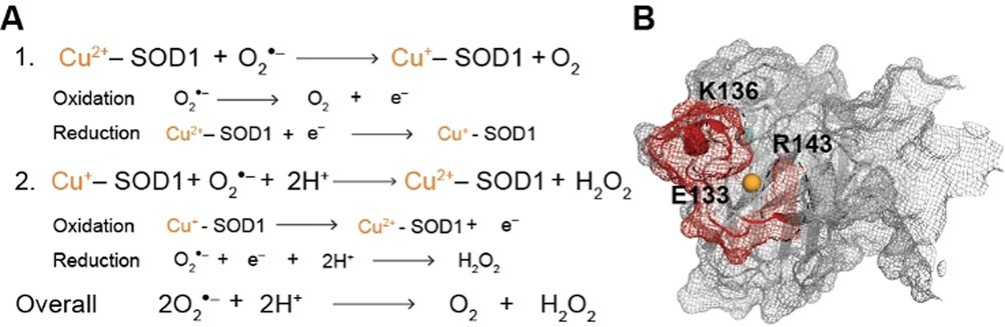
Superoxide dismutation catalyzed by SOD1. A) Redox cycling of Cu within the active site of SOD1 enables sequential superoxide (O_2_^.−^) oxidation to molecular oxygen (O_2_; reaction 1) and O_2_^.−^ reduction to hydrogen peroxide (H_2_O_2_; reaction 2). B) Charged and polar residues (red), especially within the electrostatic loop, in the active site channel provide electrostatic guidance for O_2_^.−^ towards the catalytic Cu co‐factor (orange). Lys136 (K136) and Glu133 (E133) direct the long‐range approach of O_2_^.−^, whereas Arg143 (R143) is important in hydrogen bonding with incoming O_2_^.−^ and limits incoming anion size.

Human SOD1 constitutes a frontline defence mechanism against oxidative stress, catalysing O_2_^.−^ breakdown through redox cycling of Cu bound within its active site (Figure 5 A). O_2_^.−^ is oxidised by Cu^2+^ to produce molecular oxygen (O_2_). Subsequent reduction of a second O_2_^.−^ molecule by Cu^+^ in the presence of two protons (H^+^) produces hydrogen peroxide (H_2_O_2_), which is further metabolised into H_2_O and O_2_ by catalase. SOD1 therefore constitutes a superoxide oxidase and reductase, explicable by the redox potentials involved in each half reaction (Figure 5 A; (pH 7.5, 0.2 M salt, 22 °C).^[68]^ The conformation of holo‐SOD1 protein facilitates its antioxidant function; whilst the active site and the channel leading towards it are positively charged, the remaining 89 % of the total exposure surface is negatively charged,^[69]^ creating a charge gradient that guides O_2_^.−^ towards the active site (Figure 5 B). The surface of the active site channel is formed by 18 solvent‐exposed residues, constituting approximately 11 % of the total exposed surface area (≈660 Å^2^).^[36]^ Amongst these, electrostatic loop residues Lys136 and Glu133 are particularly important in directing the long‐range approach of incoming O_2_^.−^.[^25b^, ^36^, ^70^] Arg143 also dictates O_2_^.−^ orientation within the active site channel, and combines with Thr137 to limit the size of anions approaching the Cu centre.[^25c^, ^71^] Regulation of SOD1 antioxidant activity can occur via alterations to these and other key charged residues. Acetylation^[72]^ or succinylation^[73]^ of Lys122 within the electrostatic loop reduces its net charge from +1 to 0 or −1, respectively, impeding electrostatic guidance of anionic O_2_^.−^ towards the active site. These modifications are removed by SIRT5,^[72]^ suggesting a similar regulatory role to SIRT1 in maintaining SOD1 catalytic activity. Glycation of electrostatic loop residues Lys122 and Lys128 has also been reported in human SOD1 protein,^[74]^ and may alter electrostatic guidance of O_2_^.−^ to impair SOD1 enzymatic activity when initial adducts react further to form anionic advanced glycation end products.

**Figure 6:**
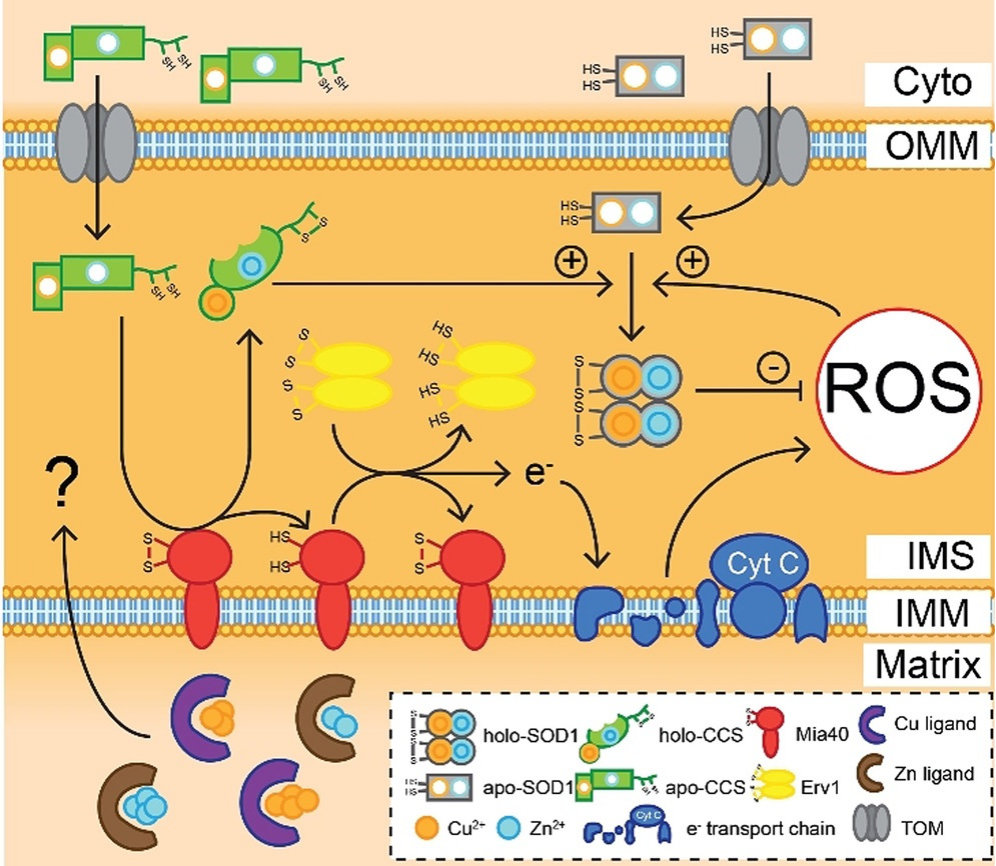
SOD1 and CCS transport and retention within the mitochondrial intermembrane space (IMS). Apo‐CCS and apo‐SOD1 protein import into the mitochondrial IMS from the cytoplasm (cyto) is facilitated by translocator of the outer membrane (TOM). The Mia40/Erv1 disulfide relay system is thought to trigger apo‐CCS maturation through disulfide bond oxidation (2SH→SS) and potentially metalation of apo‐CCS within the IMS, trapping it within this compartment. Holo‐CCS and oxidative stress work together to trigger SOD1 maturation within the IMS, mitigating the build‐up of reactive oxygen species (ROS) in this compartment produced primarily by the electron transport chain. IMM: inner mitochondrial membrane; Cyt C: cytochrome C.

As SOD1 is primarily cytosolic, its primary function is to quench cytosolic O_2_^.−^ expelled from mitochondria or produced by redox reactions involving molecular oxygen, including O_2_ binding to haemoglobin in red blood cells. Apo‐SOD1 may also cross into various subcellular compartments,^[75]^ including mitochondria, lysozomes, the nucleus and the endoplasmic reticulum.^[76]^ Given variability in pH between these compartments (pH 4–8), SOD1 is therefore considered electrostatically unusual, existing within compartments with pH values above and below its theoretical isoelectric point of 5.3. Its ability to do so is likely largely due to its ability to regulate its net negative charge across subcellular pH, only changing by ≈3 units from pH 5–8.^[77]^ Palmitoylation may be an important post‐translational modification targeting SOD1 to specific subcellular destinations by anchoring SOD1 to organellar membranes, with Cys6 palmitoylation implicated in SOD1 nuclear localisation and antioxidant activity.^[78]^ The process of mitochondrial apo‐SOD1 import and retention is regulated by CCS, translocase of the outer mitochondrial membrane and the Mia40/Erv1 disulfide relay system (Figure 6).[^76^, ^79^] Immature SOD1 and CCS are translocated from the cytosol into the mitochondrial intermembrane space by translocase of the outer mitochondrial membrane, where they both become trapped upon metal insertion and disulfide bond formation. For CCS this may be facilitated by Mia40 anchored to the inner mitochondrial membrane, as Mia40 reduction mediates CCS disulfide bond formation.[^79^, ^80^] Mia40 is reoxidised by Erv1, which donates an electron to cytochrome c for use in the electron transport chain, and down‐regulation of Erv1 is associated with decreased mitochondrial SOD1 import.^[79]^ Sudden increases in mitochondrial oxidative stress trigger CCS‐dependent SOD1 maturation within the intermembrane space, trapping it within this compartment to mitigate acute and localised oxidative insult (Figure 6). Similar to other cellular compartments, sources of Cu and Zn for immature CCS and SOD1 within the IMS are unknown, however, likely involve access to metal reservoirs within the mitochondrial matrix by as‐yet‐unidentified matrix metallochaperones. This augments SOD2‐mediated O_2_^.−^ clearance within the mitochondrial matrix.^[81]^ Substantial apo‐SOD1 distributed throughout healthy cells may therefore represent a rapid antioxidant defence mechanism, bridging the delay between oxidative‐stress‐induced *SOD1* gene induction and the synthesis and activation of nascent apo‐SOD1 protein.

**Figure 7:**
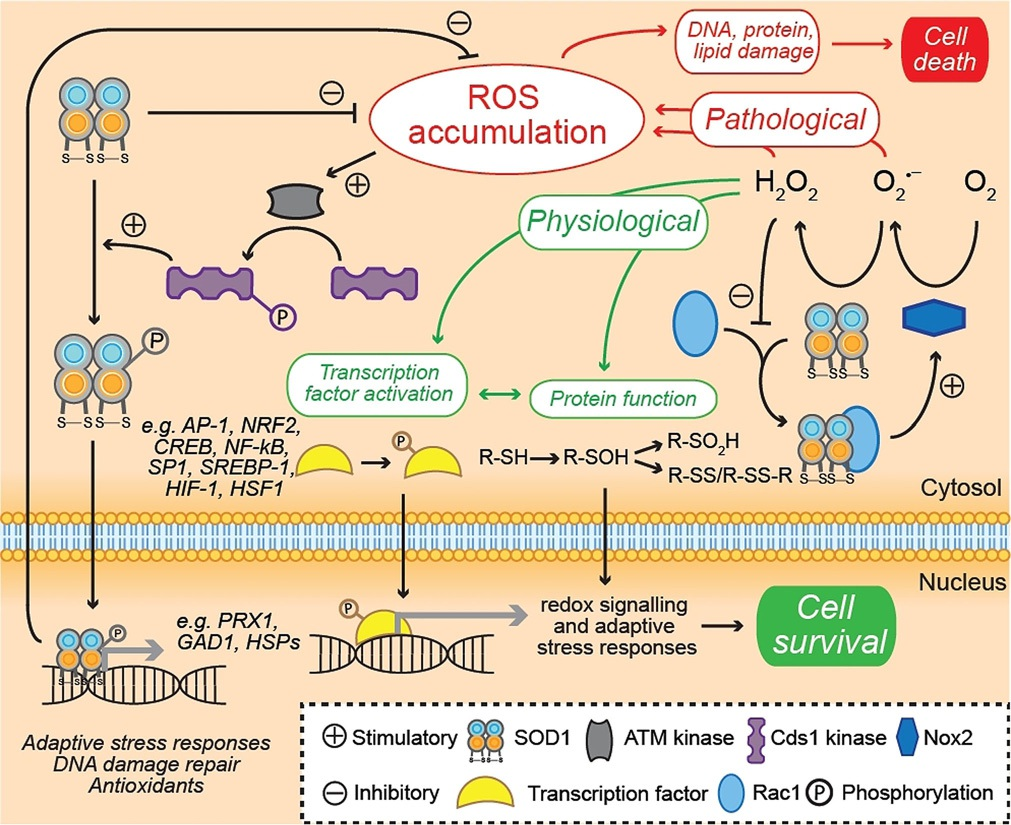
SOD1 antioxidant function and redox signaling. Aside from the enzymatic breakdown of superoxide (O_2_^.−^) radicals, SOD1 combats the accumulation of reactive oxygen species (ROS) by acting as a transcription factor for multiple genes involved in adaptive stress responses, including a large number of antioxidant genes. This occurs following phosphorylation of SOD1 by Cds1 kinase, which is itself phosphorylated and activated by ATM kinase upon increases in cellular ROS levels. By directly and indirectly influencing the levels of cellular ROS, especially O_2_^.−^ and hydrogen peroxide (H_2_O_2_), SOD1 is also an important component of redox signaling pathways governing processes such as cell proliferation and adaptive stress responses.

Additional to its principal antioxidant function, SOD1 acts as a transcription factor initiating multiple antioxidant pathways, as well as pathways governing general stress responses and DNA damage repair (Figure 7). Increases in endogenous O_2_^.−^, O_2_^.−^‐generating compounds, H_2_O_2_ and other ROS‐producing agents elicit nuclear translocation of SOD1 from the cytosol.^[6]^ Chromatin immunoprecipitation demonstrates that, once inside the nucleus, SOD1 binds to DNA promoters to regulate the expression of approximately 123 target genes (e.g. *GRE2*) involved in cellular defence against ROS, ROS‐induced DNA replication stress and DNA damage responses, general cellular stress and maintenance of cellular redox state. The relocalisation of SOD1 upon H_2_O_2_ accumulation indicates this function is not solely dependent upon O_2_^.−^, and may be a more generalised antioxidant response. SOD1 nuclear localisation may be mediated by ATM kinase and its effector Cds1 kinase, which phosphorylate SOD1 at Ser59 and Ser98 following H_2_O_2_‐dependent activation of ATM kinase,^[82]^ which is proposed to disrupt SOD1 co‐localisation with the cytoskeleton.^[83]^

A largely unappreciated secondary function of SOD1 enzymatic activity is the modulation of signal transduction pathways (Figure 7). The primary product of SOD1 catalysis, H_2_O_2_, is an important modulator of gene expression, as well as of pathways of cell proliferation, differentiation and death.^[84]^ NADPH oxygenases (e.g. Nox2) regulate H_2_O_2_ signaling by producing O_2_^.−^, which is reduced to H_2_O_2_ spontaneously or by SOD1. In addition to this downstream action of SOD1 in Nox signaling, SOD1 may act as an upstream modulator of Nox function, through a direct interaction with Rac1.^[7]^ SOD1‐bound Rac1 promotes Nox2 activation, resulting in O_2_^.−^ production. Paradoxically, when this O_2_^.−^ is then metabolised by SOD1 into H_2_O_2_ it creates a negative feedback loop regulating Nox2 function, with H_2_O_2_ inducing the dissociation of the SOD1/Rac1 complex to inhibit Nox2 activity. In this way, reactants and products of SOD1 catalysis constitute a dynamic redox signal controlling the function and signaling of specific proteins, much like other post‐translational modifications (e.g. phosphorylation). Thus, rather than considering SOD1 antioxidant activity as simply a means of removing O_2_^.−^, it should be considered a regulator of O_2_^.−^ and H_2_O_2_ levels, producing specific redox signals for desired responses to environmental stimuli.^[85]^ For example, mTORC1 regulates SOD1 activity in response to nutrient availability via phosphorylation of Thr39,^[86]^ a residue located at the entrance of the active site channel. Phosphorylation impedes anionic diffusion to the active site, reducing SOD1 activity and modulating cellular ROS. This ensures adequate cell proliferation under favourable nutrient‐rich conditions, whilst minimizing oxidative stress during nutrient deficiency. A similar increase in Ser38 phosphorylation is observed under hypoxic conditions in yeast SOD1 (Thr39 in human SOD1), and is proposed to explain why cellular antioxidant capacity is diminished under hypoxic conditions.^[87]^ Importantly, mTORC1 signaling is suppressed under hypoxic conditions,^[88]^ implicating additional kinases.

## 7 Origins of SOD1 Protein Misfolding and Aggregation

A protein is considered to be correctly folded if it assumes a fully functional and regular structure. A misfolded protein, on the other hand, exhibits substantial deviation from this optimal conformation, as a result of reorganisation or alteration of its native structure. In the specific context of SOD1, protein misfolding compromises protective antioxidant function^[89]^ and facilitates atypical molecular interactions, both between multiple misfolded SOD1 units (aggregation) and between misfolded SOD1 and other cellular constituents. In addition to unequivocal evidence of SOD1 misfolding and aggregation in *SOD1*‐linked familial ALS,^[90]^ post‐mortem analyses of sporadic ALS,^[90b]^ non‐*SOD1*‐linked familial ALS,^[90c]^ Parkinson's disease^[13]^ and Alzheimer's disease^[91]^ patient tissues suggest SOD1 misfolding may constitute a shared pathological feature amongst numerous neurodegenerative diseases. As such, formulating a more complete understanding of the molecular mechanisms underlying SOD1 misfolding and accumulation may enhance our understanding of the aetiology of these disorders and direct the development of therapies to slow or halt their progression.

The extremely high stability of holo‐SOD1 has led many[^31^, ^34^, ^92^] to speculate that SOD1 misfolding and aggregation involves intrinsically unstable apo‐SOD1. Structural characterisation of apo‐SOD1 using NMR spectroscopy,[^33a^, ^33b^] X‐ray crystallography,^[93]^ hydrogen/deuterium exchange,^[94]^ in addition to bioinformatics and computational biology approaches (PONDR; http://www.pondr.com) reveals structural instability in two specific microenvironments within apo‐SOD1—the metal‐binding and electrostatic loops—which substantially impairs the formation of a tight and functional homodimer. Whilst this instability is negated by metal binding and disulfide formation under normal cellular conditions, the incorporation of additional destabilising factors during aging or disease (*SOD1* mutations, atypical post‐translational modification of residue side chains) can disrupt the incorporation of stabilizing factors and exacerbate instability within the metal‐binding and electrostatic loops of apo‐SOD1, or perturb stabilising factors within the mature holo protein, generating partially or completely misfolded SOD1 protein.

### 7.1 Mutations

**Figure 8:**
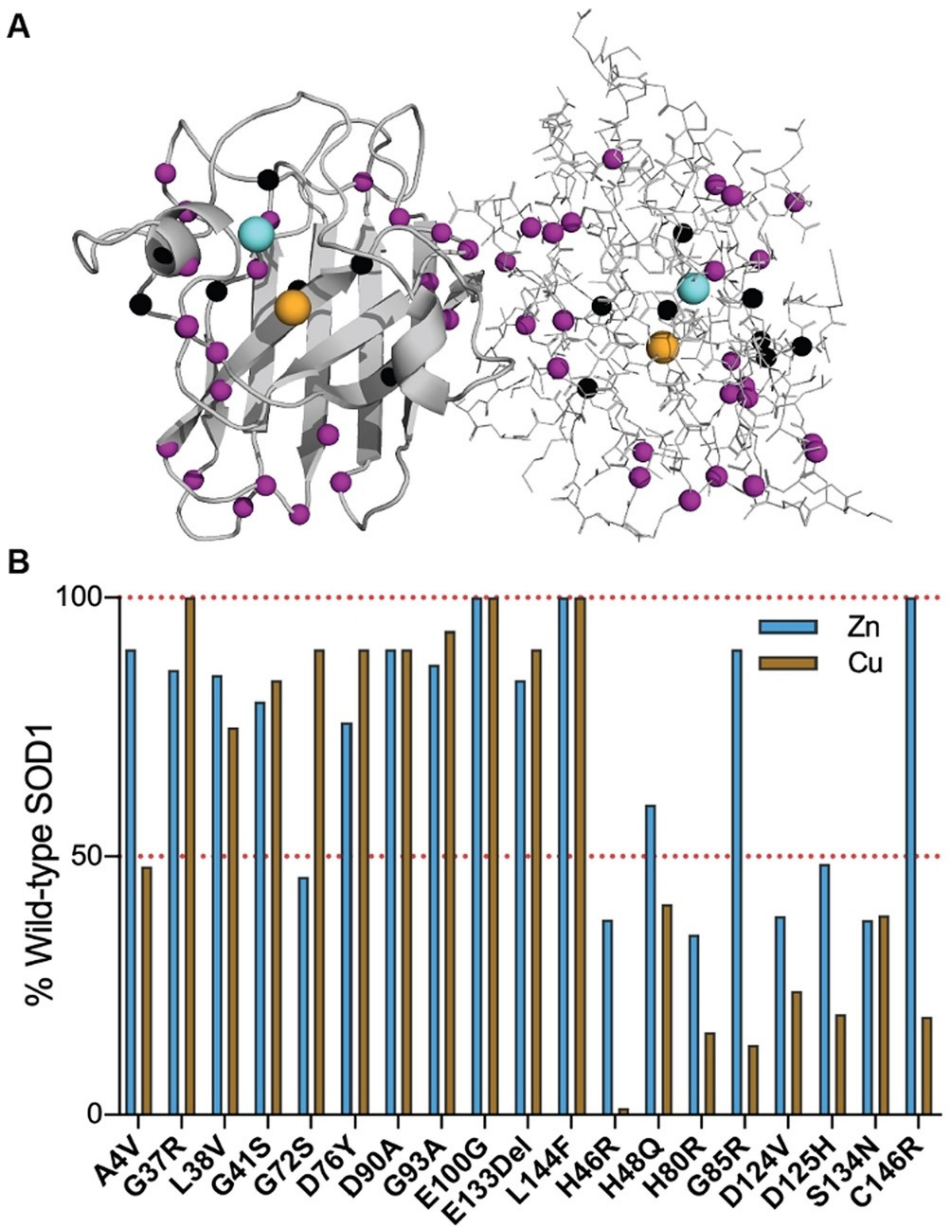
Distribution of 46 well‐studied wild‐type‐like (purple; A4V–L144F) and metal‐binding region (black; H46R–C146R) *SOD1* mutations (A) and, where known, their effect on Cu and Zn binding (B). Corresponding biochemical data for all mutations detailed in Table 2. Mutant metal binding values were averaged for mutants where metal binding data was available from more than one expression system.

Approximately 200 mutations have been documented throughout coding and non‐coding regions of the *SOD1* gene (http://alsod.iop.kcl.ac.uk),[^12^, ^95^] primarily comprising amino acid substitutions (>80 %), as well as insertions, deletions and genetic polymorphisms. The structural and functional consequences of these diverse mutations are continually being studied in vitro using common human protein expression systems, such as yeast^[96]^ and Sf21 insect cells,^[97]^ as well as in higher organisms including mice^[98]^ and non‐human primates.^[99]^ From these data, SOD1 mutants are typically segregated into two broad groups: metal‐binding region (MBR) mutants which exhibit impaired metal binding and diminished catalytic rates, and wild‐type‐like (WTL) mutants which possess more similar metal‐binding affinities, structural conformations and enzymatic activities to wild‐type SOD1 protein (Table 2; Figure 8). Significant discrepancies in metal binding, enzyme function and protein stability further subdivide mutant proteins within MBR and WTL groups, confounding the search for a unifying molecular disturbance between SOD1 mutants that may underlie misfolding. Decades of research in pursuit of such a singularity has, however, uncovered common themes between select subgroups of mutant proteins, which may collectively be predictive of the propensity for all mutant proteins to misfold.^[100]^

**Table 2:** Biochemical data and corresponding *SOD1*‐linked familial ALS patient survival times for 58 mutant SOD1 proteins.

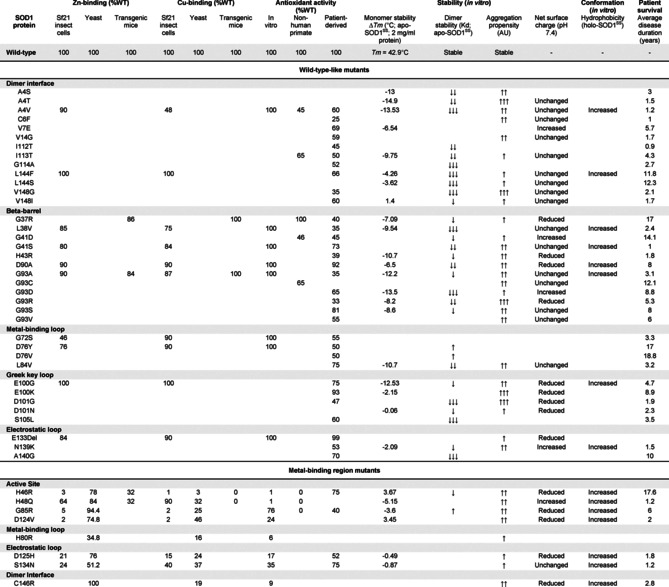

Unsurprisingly, abundant data demonstrate that MBR mutations promote the accumulation of immature metal‐deficient SOD1 metalloforms, with mutant proteins frequently isolated from expression systems containing little to no bound metals (Table 2).[^96a^, ^97a^, ^98a^] Whilst MBR mutations dramatically impair holo‐SOD1 formation and stability, most are of little consequence to apo‐SOD1 stability, with some mutant apo proteins (H46R, D124V) even exhibiting increased thermodynamic stability relative to wild‐type apo‐protein (Table 2).^[96a]^ It is understandable that the structural consequences of MBR mutations are largely masked in apo‐SOD, given that intrinsic disorder within the apo protein is primarily localised to regions containing metal‐binding residues, in addition to regions such as the electrostatic and disulfide loops. This does not imply MBR mutations are benign; on the contrary, most MBR SOD1 mutants possess an increased propensity for self‐assembly.^[101]^ The primary consequence of these mutations is the prevention of SOD1 maturation, yielding intrinsically disordered, aggregation‐prone immature SOD1 conformers, which are vastly more susceptible to misfolding in situations of even mild cellular stress.[^31^, ^102^] In the instance of some of MBR mutations, disruption of the native Zn‐binding site may trap Zn within the folding nucleus (Cu ligands),^[103]^ triggering aggregation of these immature SOD1 metalloforms.^[104]^

In contrast to MBR mutants, WTL mutants are typically isolated from expression systems exhibiting higher metal occupancies[^96a^, ^97^, ^98^] and enzymatic activities compared to MBR mutants (Table 2; Figure 8).[^97a^, ^99a^] Their primary destabilizing influence is often more apparent upon the removal of bound metal ions; almost all WTL mutant apo proteins demonstrate substantial impairment of apo‐dimer formation[^102c^, ^105^] and are unfolded at, or close to, physiological temperature.[^31^, ^96a^, ^106^] These factors are collectively likely to underlie their increased aggregation propensity (Table 2).[^101^, ^106^] A number of investigated WTL mutants (A4V, L38V, G93A)^[107]^ also display altered metal‐binding kinetics, particularly at the native Zn site.^[108]^ These findings attest to the structural importance of specific residues outside metal coordination sites for SOD1 metal binding, which may not ligate metals themselves but can modulate protein conformation around coordinating residues to influence metal binding. Accordingly, a number of isolated WTL mutants (A4V, L38V, G41S, G72S, D76Y) are unable to retain full metal occupancy in vitro (Table 2). In‐cell NMR spectroscopy further reveals that a range of WTL mutants (A4V, I35T, G37R, G93A, I113T) exist predominantly as unstructured apo‐proteins unable to bind zinc when over‐expressed in human HEK293T cells, or when expressed and purified from *E. coli*.^[43a]^

Taken together, the molecular consequences of MBR and WTL *SOD1* mutations appear to merge into a single unifying theme underlying SOD1 protein misfolding: mutations not only disrupt SOD1 maturation to promote the accumulation of disordered immature SOD1 conformers, but may substantially accentuate structural instability within already disordered microenvironments in apo‐SOD1. Multiple studies conducted under physiological conditions (temperature, pH, and protein concentration) have identified exacerbated instability within the dimer interface[^105a^, ^109^] and/or the electrostatic loop[^35^, ^110^] of the holo and apo proteins of numerous MBR and WTL mutants. Seventy out of seventy‐five investigated SOD1 mutations (MBR and WTL) result in dimer instability and/or increased dimer dissociation in silico,^[111]^ resulting from widespread disruption of hydrogen bonding networks in β‐strands 1, 2, 7, and 8, the dimer interface, and the electrostatic, disulfide, and Greek key loops.^[39b]^ The degree of destabilisation does, however, vary considerably between individual mutants. The A4V mutation introduces a larger hydrophobic residue into the SOD1 dimer interface to substantially disrupt hydrogen‐bonding networks, whereas a select few alternative mutant proteins (H46R, N86S, D90A, E100K, D101N, L117V, N139K, V148I) are barely distinguishable from wild‐type SOD1 when comparing apo protein stability, dimer dissociation and/or metal coordination (Table 2).[^96a^, ^105b^, ^105c^, ^106^] As with other mutants,[^101^, ^106^] many of these comparatively stable mutations result in an increased propensity for aggregation in transgenic mice, in cell culture or in vitro, compared with wild‐type SOD1.^[116]^ The high aggregation rate of these mutant species may involve aggregation from a native‐like holo state,^[113a]^ whereby mutations increase surface hydrophobicity or lower net protein charge from 7.37(±0.05) per dimer at physiological pH (7.4),[^77^, ^117^] promoting the formation of native‐like oligomers that undergo misfolding similar to that normally preceding aggregation. This aggregation mechanism may in fact even play a role in the aggregation of more unstable SOD1 mutants, compounding aggregation originating from immature misfolded apo metalloforms. Accordingly, D90A, E100K, D101N and N139K SOD1 mutants, together with most other SOD1 mutants, exhibit either a reduced net charge,[^97b^, ^113a^, ^118^] increased surface hydrophobicity[^97b^, ^115^] or both, albeit to different extents (Table 2). This is especially relevant for mutants whose greatest electrostatic effect is observed in the mature protein (E100K), where net charge is lowered by up to 50 %.^[119]^ It is important to remember, however, that charge alterations associated with various *SOD1* mutations will differ depending on the pH of the subcellular compartment within which SOD1 resides, as well as the maturation/metalation state of SOD1 within that compartment.[^77^, ^119^] These biophysical changes are consistent with the exposure of hydrophobic residues located in the SOD1 dimer interface, amongst other conformational changes, and may be indicative of consistent dimer instability. Higher dimer dissociation constants have been measured for most mutant proteins compared to wild‐type SOD1 (Table 2), including the most stable mutations.^[111]^ These measurements, however, are currently unavailable for some of the most stable SOD1 mutants (L117V), and represent important areas for future investigation given they may underlie misfolding or aggregation of these more perplexing mutant proteins.

Apo‐protein destabilisation is often considered insufficient to explain mutant SOD1 misfolding and aggregation, given mutant apo‐SOD1 stability is virtually unchanged (D101N, D125 H, S134N) or improved (H46R, D124V, V148I) for a number of WTL and MBR mutations compared with wild‐type apo protein in vitro.[^96a^, ^106^] This conclusion, however, is based upon data derived from comparatively simple and tightly controlled experimental environments, where mutations are assumed to be the only destabilizing influence. Some mutations may indeed impart no destabilizing influence on apo‐SOD1 per se, but may promote misfolding and aggregation in more complex biological systems by exposing greater quantities of disordered immature SOD1 conformers to additional destabilizing influences in vivo, including atypical post‐translational modifications to key residue side chains.

### 7.2 Aberrant Post‐translational Modifications

**Figure 9:**
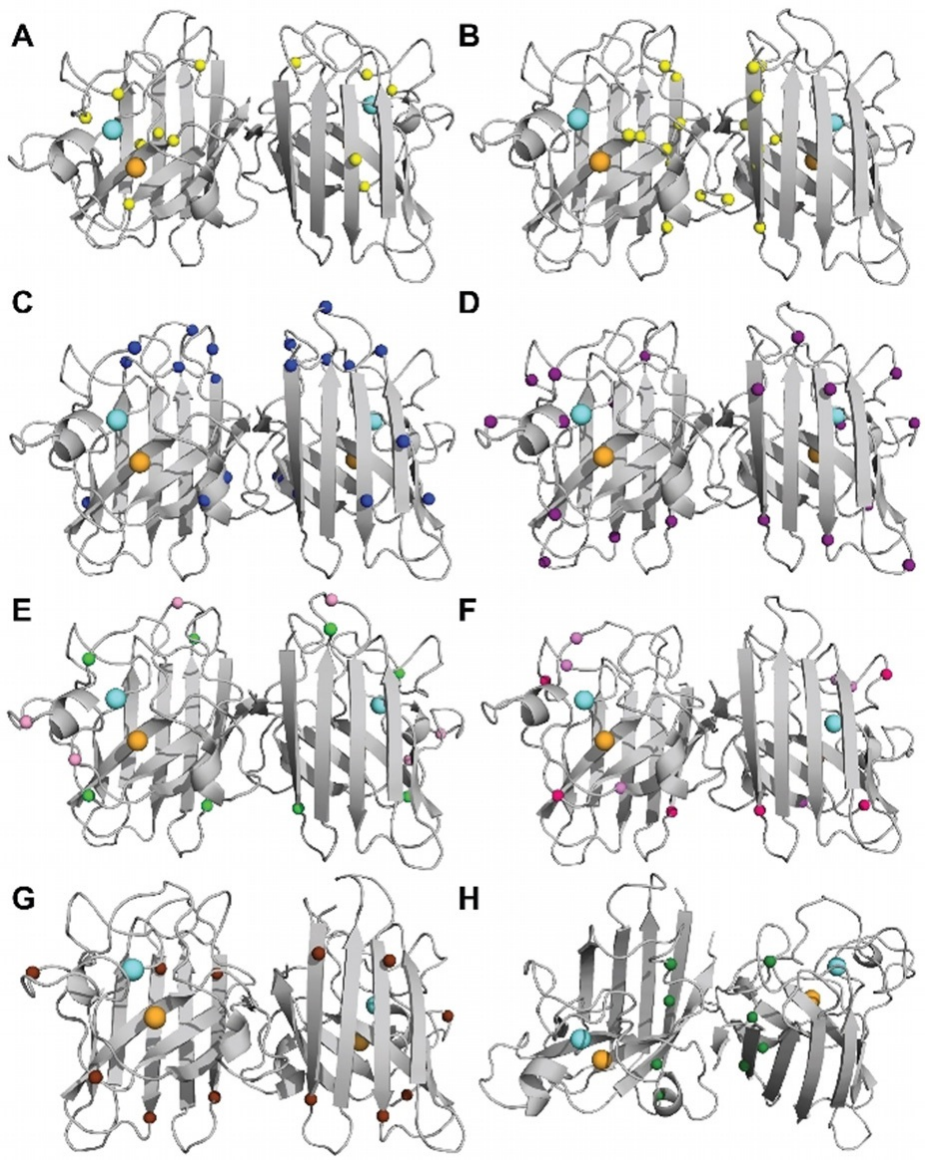
Distribution of known sites of post‐translational modification for human SOD1 protein. Included: oxidation (A, B; yellow), phosphorylation (C; blue), ubiquitylation (D; purple), acetylation (E; light green), deamidation (E; light pink) succinylation (F; dark pink), methylgloxalation (F; violet), glycation (G; brown), palmitoylation (H; dark green). Residues labeled in all panels were identified from analyses of human SOD1 protein using mass spectrometry (PhosphoSitePlus^®^, Cell Signalling Technology),[^102a^, ^120^] with the exception of panel B which was performed in silico.^[121]^ Specific residue numbers listed in Supplementary Table 1.

As previously discussed, the physiological structure and function of SOD1 is regulated by key post‐translational modifications (Supplementary Table 1): metal binding and disulfide bond formation (Cys57, Cys146) improve structural stability, phosphorylation (Ser60, Ser99) triggers nuclear localisation, ubiquitylation (Lys63) regulates degradation, and acetylation (Lys70, Lys122), phosphorylation (Thr39), glycation (Lys122, Lys128) and succinylation (Lys122) may play roles in regulating protein maturation and antioxidant activity. Alterations in the levels of these modifications, as well as the presence of atypical modifications on certain residues, are associated with mutant and wild‐type SOD1 misfolding (Figure 9).

Irregular post‐translational modification of specific holo‐SOD1 residues can promote dimer dissociation by dislodging stabilizing metal co‐factors or disrupting important charge interactions across the dimer interface. Oxidative modifications, in particular, trigger destabilisation and aggregation of wild‐type and mutant SOD1 under physiological conditions in vitro (Figure 9 A).[^121^, ^122^] Metal‐coordinating histidine ligands (His46, His63, His71, His80, His120) are particularly sensitive to H_2_O_2_‐induced side‐chain oxidation in vitro[^120c^, ^122b^] due to their nucleophilic aromatic side chain,^[123]^ resulting in substantial Cu (<70 %) and Zn (<30 %) dissociation. Increased protein hydrophobicity, as measured by 1‐anilinonaphthalene‐8‐sulfonate (ANS) fluorescence,^[122b]^ further indicates loosening of the dimer interface in these oxidised species. Such mild‐moderate structural flexibility is thought to be sufficient to allow solvent access to residues normally buried within the protein's hydrophobic core (Phe20)^[122c]^ and dimer interface.^[121]^ Subsequent oxidation (carbonylation) of dimer interface residue side chains (Lys3, Lys9 and Arg115) significantly compromises dimer stability in silico (Figure 9 B), by disrupting key hydrogen‐bonding and charge interactions across the interface.^[121]^ For interface residues such as Lys3 and Lys9, side‐chain oxidation exerts a destabilizing effect that is equally as potent as any known *SOD1* mutation in silico, indicating that two or more oxidation events are capable of triggering dimer dissociation.^[121]^ Further investigations are warranted to confirm the translation of these findings in vitro or in vivo; however, difficulties arise in directing side‐chain oxidation to specific SOD1 residues. In addition to multiple compounding side‐chain oxidation events, the introduction of common *SOD1* mutations (L38V, G85R, G93A, and I113T) can augment SOD1 misfolding and aggregation induced by side‐chain oxidation,[^122b^, ^122c^] provided mutations do not disrupt side‐chain solvent accessibility. Structural flexibility within some mutant proteins may therefore similarly increase solvent accessibility of oxidation‐prone side chains normally buried within the protein, whose oxidation can trigger misfolding and aggregation. Aside from metal‐binding residues, oxidation of solvent‐accessible Trp32 and glutathionylation of Cys111 both trigger dimer dissociation and aggregation.[^110^, ^122a^, ^124^] Trp32 is found at the core of a segment of SOD1 protein (residues 28–38) that is heavily implicated in SOD1 oligomer formation,^[125]^ and oxidation of its indole side‐chain ring system is shown to play a role in triggering self‐assembly of SOD1. Cys111 is found within the Greek key loop, itself heavily involved in the complex hydrogen‐bonding network stabilizing the dimer interface.

As the addition of post‐translational modifications to any protein is highly dependent upon residue solvent exposure, it is unsurprising that apo‐SOD1 has a much higher propensity for aberrant modification than the holo protein. Accordingly, exposure of purified apo‐ and holo‐SOD1 proteins to mild oxidative stress at physiological temperature (37 °C), pH (7) and protein concentration (100 μM) results in amino acid side‐chain oxidation and aggregation of apo protein only.^[126]^ Inherent structural disorder within the apo protein promotes oxidative modification of cysteine, lysine and arginine residues, in particular, whose side chains are susceptible to oxidation due to their high nucleophilic properties^[127]^ and are otherwise protected by dimerisation. Oxidation of Lys3, Cys6, Lys9, Thr54, Cys111, Arg115 and Thr116 residue side chains substantially destabilises apo‐SOD1 in silico,[^34^, ^102a^, ^121^, ^122c^, ^126^] which likely impairs dimer formation and triggers misfolding. Conversion of the sulfhydryl groups (Cys‐SH) of Cys6 and Cys111 to sulfonic acid (Cys‐SO_3_H) constitutes especially important oxidative modifications to apo‐SOD1 in vitro; hydrogen‐bonding participation triples due to additional hydrogen‐bonding acceptor sites in Cys‐SO_3_H, and net charge increases significantly.^[121]^ The threefold increase in interaction energy between oxidised cysteine residues and their surrounding residues likely mediates structural deviation of Cys‐oxidised apo‐protein from its native conformation in silico, and may play a significant role in SOD1 self‐assembly.^[128]^ Indeed, mass spectrometry identifies Cys6 and Cys111 of apo‐SOD1 as primary targets of oxidative stress in vitro and in vivo, which promotes oligomerisation under physiological conditions.^[126]^ Past oxidative modifications, glycation of electrostatic loop residues disrupts intramolecular bonding networks to promote apo‐protein misfolding.^[129]^ Methylglyoxal, a highly reactive by‐product of glycolysis, has also recently been demonstrated to preferentially react with arginine residues (Arg69, Arg79, or Arg143) within apo‐ and Zn‐bound‐SOD1 metalloforms.^[130]^ This favors monomerisation and unfolding, and may promote the formation of a high number of subsequent destabilising glycations involving additional lysine and cysteine residues. Site‐directed mutagenesis of asparagine residues at positions 26, 131 and 139 to aspartate, mimicking deamidation, greatly reduced the stability of the holo‐SOD1 dimer compared with wild‐type SOD1, attributed to a destabilisation of the electrostatic loop.^[131]^ As expected, deamidation also significantly increased the aggregation propensity of SOD1 to levels comparable to N139D mutant SOD1 protein. These residues are predicted to undergo significant deamidation (>20 %) on time scales comparable to the long lifetime (>1 year) of SOD1 in large motor neurons. Capillary electrophoresis and mass spectrometry further demonstrate that ≈23 % of Asn26 is deamidated to aspartate in wild‐type human SOD1 isolated from human erythrocytes,^[131]^ suggesting that the high solvent exposure of the Ser25‐Asn26‐Gly27 motif within loop II of SOD1 may predispose N26 to spontaneous deamidation.

Aside from these examples, little is known about the biophysical impact of many other site‐specific post‐translational modifications on apo‐SOD1 stability, despite numerous solvent‐accessible sites for glutathionylation, acetylation, glycation, phosphorylation, succinylation and ubiquitylation (Figure 9).[^62^, ^72^, ^86^, ^120b^, ^124^] Advances in this area will require greater refinement of emerging methodologies designed to target certain modifications to specific protein residues in vitro and in vivo,^[132]^ including selective chemical modification of proteins, protein semisynthesis, native chemical ligation, and the genetic non‐canonical amino acid incorporation approach.^[133]^

### 7.3 Altered Metalation—Availability and Delivery

Aside from direct alteration of SOD1 protein, alterations to pathways of metal delivery to SOD1 may feasibly promote SOD1 mismetalation and misfolding. Cu turnover in the central nervous system is much slower than within other peripheral organs, even under physiological conditions, and hence maintaining an equilibrium of bioavailable Cu is particular important for metalloproteins in the brain and spinal cord.^[134]^ Cu incorporation into SOD1 requires Ctr1‐mediated cellular Cu import,^[135]^ and Cu chaperoning to SOD1 via CCS‐dependent[^54^, ^136^] and independent^[57]^ mechanisms, as discussed in Section 5 of this review (Figure 3). Cu‐deficient conditions up‐regulate levels of Ctr1 and CCS proteins to ensure adequate Cu supply and delivery to SOD1; however, this may be impaired following alteration of cysteine/methionine Ctr1 Cu ligands,^[137]^ depletion of CCS‐independent SOD1 cuprochaperones,[^51^, ^55^] or by disruption of numerous CCS structural motifs including:[^51^, ^138^] the MXCXXC Cu‐binding motif (D1), Zn‐binding residues (His147, Asp167; D2),^[46]^ SOD1 recognition sequence (D2),^[139]^ and lysine/arginine residues implicated in CCS membrane association and Cu acquisition from Ctr1.^[140]^ C‐terminal modification of Ctr1 may also disrupt the Ctr1‐CCS interaction at the plasma membrane and promote Ctr1 endocytosis and degradation.[^135^, ^141^] Dysregulation of Sp‐1, a Zn‐finger transcription factor that acts as a Cu sensor to modulate Ctr1 cell‐surface expression, may also influence the levels and localisation of Ctr1.^[142]^ By contrast, disruption of ATP7A‐mediated Cu efflux^[143]^ does not affect SOD1 protein levels or activity,^[144]^ suggesting cytoplasmic Cu accumulation does not influence SOD1 expression to the same extent as Ctr1‐dependent Cu deficiency. This is particularly interesting given that mutant SOD1 reportedly promotes progressive spinal cord Cu accumulation in hSOD1^G93A^ mice via up‐regulation of Ctr1, CCS, Atox1 and Cox17 cuproprotein expression, and down‐regulation of ATP7A.^[145]^

Little is known regarding the specific contribution of glial Cu homeostasis to neuronal SOD1 metalation; however, astrocytes possess a greater Cu storage capacity and supply Cu to neurons.^[146]^ Astrocytes are also comparatively resilient to Cu toxicity, most likely as a result of their capacity to store excess Cu complexed to glutathione and metallothioneins.^[146]^ Severe alterations in the Cu storage capacity of glia, mostly likely via alterations in glutathione or metallothioneins, may therefore influence neuronal Cu availability and SOD1 metal acquisition. It must be recognised, however, that SOD1′s comparatively strong Cu‐binding affinity may dictate its preferential metalation over other neuronal cuproproteins in environments of reduced Cu.^[63]^ Changes in SOD1‐specific Cu binding mechanisms, such as SOD1 Cu coordination or CCS‐SOD1 recognition and Cu transfer, may therefore be more realistic threats to physiological SOD1 metalation.

Much less is known regarding Zn import, chaperoning and incorporation into SOD1, and therefore it is very difficult to even speculate as to the biochemical changes that might impede physiological SOD1 metalation. Alterations in neuronal Zn import arising from disruptions to Zrt‐/Irt‐like proteins (ZIPs),^[147]^ neuronal and glial Zn storage within metallothioneins,^[148]^ and Zn export through ZnT Zn transporters,^[149]^ could similarly theoretically impact SOD1 metal availability; however, as per Cu, the high Zn‐binding affinity of SOD1 designates it as a priority target for Zn delivery.^[150]^ Recent evidence for a molecular chaperone roll for CCS in promoting SOD1 Zn‐binding^[43a]^ may provide a mechanistic basis for reduced Zn‐binding by some WTL SOD1 mutants expressed in cell culture and transgenic mice (Table 2). Just as impaired SOD1 Cu‐binding may result from alterations to Cu‐binding or SOD1 recognition motifs within Ctr1 or CCS proteins, so too may SOD1 Zn‐binding following genetic mutations or chemical alterations to important residue side chains within CCS protein promoting SOD1 Zn acquisition. Noteably, co‐expression of human CCS rescues impaired Zn‐binding capacity of a range of WTL mutant SOD1 proteins (A4V, V7E, G37R, T54R, G93A, I113T, V148I) expressed in human HEK293T cells, suggesting these mutations do not alter SOD1‐CCS coupling.^[43a]^

Just as the destabilizing influences of aberrant post‐translational modifications to SOD1 protein have traditionally been eclipsed by research focussing on *SOD1* gene mutations, we highlight that disruptions to pathways of physiological SOD1 metal delivery, metalation and maturation may be a significant contributor to SOD1 misfolding, warranting investigation.

### 7.4 Current Approaches Investigating SOD1 Metalation and Misfolding: Strengths and Limitations

Altered SOD1 metalation can be argued to drive SOD1 misfolding; nearly all investigated *SOD1* mutations and atypical protein modifications diminish SOD1 metal content relative to wild‐type protein (Table 2). These data are generated using numerous highly feasible human protein expression systems (*S. cerevisiae*, Sf21 insect cells, murine models) and protein purification methodologies; however, these approaches are not without limitations; even wild‐type human SOD1 purified from these systems is metal‐deficient, particularly with regards to Cu (≈0.3–0.5 equivalents/dimer).[^96a^, ^97^, ^98^] It is therefore important to clarify whether mismetalation of mutant or modified SOD1 proteins derives from isolation and quantification protocols, expression systems and/or destabilizing factors.

Immunocapture and hydrophobic interaction chromatography are routinely used for extracting human SOD1 proteins from common expression systems, followed by measurement of total extract metal levels using inductively coupled plasma‐mass spectrometry (ICP‐MS).[^96a^, ^97^] SOD1 exists within cells as a mixture of different metalloforms, so it is unsurprising that metal equivalents per unit of protein are frequently quantified below full occupancy using these methods;[^96a^, ^97^, ^98^] isolation of metal‐free (apo) and metal‐deficient metalloforms^[151]^ along with holo‐SOD1^[97a]^ dilutes total metal:protein ratios. Metal reconstitution is often employed to negate this dilemma by promoting the maturation of apo and metal‐deficient metalloforms to their holo state; however, this can substantially distort metal coordination geometry[^107^, ^152^] and prevents valuable information regarding SOD1′s native post‐translational modifications from being collected. Such approaches are therefore unable to inform whether variations in metal equivalents between mutant/modified proteins are representative of impaired apo protein metal binding, intermediary metal incorporation, or holo protein metal retention, all of which could be accurately resolved through separation and quantification of distinct SOD1 metalloforms. Beckman and colleagues^[153]^ have pioneered the development of a mass spectrometric methodology capable of just this, which they have recently successfully applied to a number of mutant SOD1‐expressing mice.[^151^, ^154^] The refinement of such methods will be key to improving the detection of SOD1 mismetalation. Aside from inherent limitations of common measurement approaches, physiological inconsistencies between human SOD1 protein and various expression systems may contribute to measured SOD1 mismetalation. For example, whilst the N‐terminal alanine of SOD1 is acetylated in human cells and yeast, this is not the case for human SOD1 expressed in *E. coli*.^[155]^ The sheer level of human protein within many of these systems (≥17‐fold elevation above physiological levels) represents a substantial deviation from physiological conditions in itself.^[156]^ Aside from overloading endogenous CCS‐dependent and ‐independent metalation pathways,^[154]^ disproportionately high SOD1 expression may create a mismatch between Cu demand and supply in the brain and spinal cord, where Cu import is much less efficient than in the periphery.[^134^, ^157^] This may contribute to SOD1 mismetalation and partially underlie the reduction in SOD1 specific activity (SOD1 enzymatic activity per unit of protein) observed in transgenic mutant SOD1 mice.^[157]^ Internal attempts made by these transgenic human SOD1 mice to rectify this mismatch are evidenced by an 8‐fold increase in their endogenous CCS expression;^[145b]^ however, the reported inadequacy of mouse CCS in completing human SOD1 maturation suggest this response falls short.^[154]^ The absence of glia in single cell culture expression systems (for example, *S. cerevisiae*, Sf21 cells) may also contribute to supply/demand imbalances, owing to the pivotal role of glia in Cu and Zn storage and supply to neurons in vivo.[^146^, ^148^] Proportionate co‐expression of human CCS in these systems was expected to partially ameliorate this mismatch; however, intriguingly the Cu content of SOD1 in hSOD1^G93A^/hCCS mice was found to be even lower than that quantified in hSOD1^G93A^ mice.^[154]^ Elevated Cu acquisition by hCCS in these double transgenic mice was also found to be at the expense of mitochondrial cytochrome c oxidase (COX) Cu loading, triggering severe mitochondrial dysfunction.^[158]^ Common simple human SOD1 expression systems do seem to recapitulate some important aspects of human SOD1 biology; human SOD1 protein expressed in *S. cerevisiae* and mouse fibroblasts, for example, engages in both CCS‐dependent and ‐independent metalation pathways,[^55^, ^159^] regardless of the finding that endogenous *S. cerevisiae* Sod1 is solely dependent upon CCS for metal incorporation and disulfide formation.^[160]^ Despite differences between endogenous SOD1 proteins, these systems are therefore seemingly capable of facilitating many aspects of human‐like SOD1 protein maturation, although it is unclear whether this occurs as efficiently as in human cells.

In other more complex biological systems, however, the evidence appears to be on the contrary. As discussed in great detail in the next section, transgenic expression of numerous human mutant SOD1 proteins elicits a robust motor neuron phenotype in mice. These mice are widely used to model *SOD1*‐linked familial amyotrophic lateral sclerosis, and to investigate how *SOD1* mutations alter SOD1 biochemistry and contribute to motor neuron degeneration in this disorder. An important caveat of these models, however, is the substantial discrepancy in disease severity and progression between humans and mice for a given mutant protein. Human *SOD1*‐linked familial ALS patients with an A4V mutation typically exhibit early onset, rapidly progressing ALS,^[161]^ whereas in mice the expression of a *SOD1* ^*A4V*^ transgene elicits a slow progressing form of ALS which develops comparatively late in life.^[156]^ The converse is also true for mutants including *SOD1* ^*G93A*^, where mice show quick and severe progression whilst disease progression in humans with this mutation is comparatively slow.^[162]^ It is unclear whether this is indicative of fundamental differences in the biochemistry of a given mutant when residing in human vs. murine nervous tissues, or simply whether mutation‐specific alterations to SOD1 biochemistry influence the rate and severity of motor neuron degeneration in humans and mice differently. Either way, these disparate disease courses between mice and humans for a given mutant protein complicate our understanding of mutant SOD1 pathology, and represent an obstacle in the utility of models for characterizing mutant protein biochemistry and recapitulating facets of human disease.

Overall, although current expression systems provide highly feasible means of studying SOD1 protein metalation and misfolding, absolute conclusions regarding the influence of destabilizing factors on human SOD1 protein metalation and biochemistry should be interpreted with caution. Variation in metalation, misfolding and activity measurements for a given mutation or modification, both between experimental systems (e.g. *S. cerevisiae* vs. Sf21 cells; Table 2), and between these systems and human tissue samples (Table 2), indicates a degree of divergence of these systems from human physiology. Future investigations may consider employing human‐derived expression systems to cross‐validate any major findings and minimise systemic deviation from physiological conditions. In particular, the use of in‐cell NMR spectroscopy in human cells possesses great potential as a means of dissecting pathways of protein maturation and post‐translational modification under near‐physiological conditions of pH, redox potential, viscosity and the presence of all relevant interaction partners.^[163]^ Successful application of in‐cell NMR spectroscopy to studying human proteins expressed endogenously in human cells relies on efficient cDNA transfection, relatively high protein expression levels, applicability of different labeling strategies and maintenance of cell integrity during measurements.^[163]^ In this regard, Banci and colleagues have pioneered the development and use of this technology in SOD1 biology, successfully addressing these methodological aspects in their use of in‐cell NMR to investigate SOD1 protein maturation.[^43a^, ^52^, ^56^] Their data profile the SOD1‐CCS interaction, as well as the impact of *SOD1* mutations on this interaction, within living human cells to a level of detail not previously possible.

Until technologies for SOD1 metalloform quantification are refined, and expression systems are made to more accurately replicate human physiology, it remains unclear whether altered SOD1 metalation in common models of mutant and wild‐type SOD1 misfolding is a product of destabilizing factors, expression systems, or isolation and quantification procedures, and thus whether SOD1 mismetalation possesses any relevance to human health or disease.

## 8 SOD1 Misfolding Is Associated with Neurodegeneration

Mutant SOD1 protein misfolding has been implicated in spinal cord motor neuron degeneration in familial ALS linked to *SOD1* gene mutations (*SOD1*‐linked familial ALS) for over 25 years.[^12^, ^113a^, ^164^] Accumulating evidence suggests wild‐type SOD1 misfolding and dysfunction may also contribute to the death of spinal cord motor neurons in sporadic ALS,[^90b^, ^110^, ^125^, ^165^] substantia nigra pars compacta (SNc) dopamine neurons in Parkinson's disease,[^13^, ^91^, ^166^] and neurons within the frontal cortex and hippocampus in Alzheimer's disease.[^91^, ^167^] Protein misfolding has dual implications; compromising normal protein function as well as forming abnormal interactions with other molecular pathways, thus potentially contributing to pathology through both loss‐ and gain‐of‐function actions.

### 8.1 Loss‐of‐Function

Mutation‐ or modification‐induced SOD1 misfolding diminishes antioxidant activity (Table 2),^[89]^ impairs nuclear translocation and promoter binding,^[168]^ and disrupts SOD1 redox signaling.^[7]^ Whilst not considered to trigger neuron death *per se*, a loss of these protective SOD1 functions sensitises neurons to degeneration induced by cellular stressors. Five *Sod1* ^*−/−*^ mouse strains have been generated to examine the consequences of SOD1 loss‐of‐function for motor neuron survival in *SOD1*‐linked familial ALS, each obtained by targeted deletion of different *Sod1* genomic sequences. Phenotypes are strikingly similar between all five strains, mice exhibiting adult‐onset progressive motor dysfunction, measured by rotarod performance, gait,^[169]^ grip strength and tremours.^[170]^ Neurophysiological investigations correlate these changes with selective muscle denervation and motor axon deficits,^[171]^ originating in the neuromuscular junction before spreading to the neuronal cell body and impacting fast‐twitch muscle fibres preferentially.^[172]^ Biochemically, the complete loss of SOD1 protein elevates mitochondrial ROS generation and promotes cellular redox dyshomeostasis, reversed by the selective replacement of SOD1 within the mitochondrial inter‐membrane space.^[173]^ Mitochondrial densities are reduced in *Sod1* ^*−/−*^ axons, which may contribute to motor axon deficits, particularly distal axons which require relatively greater energy input for maintenance. Importantly, no neurodegenerative disorder exhibits complete abolishment of SOD1, and therefore it is unreasonable to equate a complete loss of SOD1 enzymatic activity, in isolation, with the development of neurodegenerative disorders.

Perhaps more relevant, a murine model expressing a modest 50 % reduction in SOD1 protein (*Sod1* ^*+/−*^) elicits modest motor dysfunction and increases neuronal susceptibility to degeneration following axotomy^[174]^ and other cellular stressors such as glutamate toxicity,^[175]^ cerebral ischemia,^[176]^ and aging.^[177]^ An increase in blood–brain barrier permeability was observed in these animals,^[176]^ suggesting a dose‐dependent relationship between SOD1 deficiency and neuronal susceptibility to oxidative insult. These data possess clear relevance to the aetiology of *SOD1*‐linked familial ALS, where a modest reduction in SOD1 activity is documented within vulnerable motor neurons for a variety of *SOD1* mutations (Table 2),^[89]^ suggesting reductions in SOD1 function may underlie a number of abnormal motor neuron phenotypes in this disorder which increase their susceptibility to injury. The potential for wild‐type SOD1 dysfunction to contribute to sporadic ALS aetiology is less clear; SOD1 activity is unchanged in red blood cell lysates^[178]^ and post‐mortem frontal cortex^[179]^ of sporadic ALS patients, although to our knowledge activity levels have not been quantified in regions of the spinal cord or brain exhibiting severe motor neuron degeneration in sporadic ALS, with such investigations warranted.

Importantly, motor neurons are not the only neuronal population whose high metabolic demands predispose them to oxidative stress. Calcium pacemaking activity^[180]^ and a large, unmyelinated axonal arbour^[181]^ impose a high metabolic demand upon SNc dopaminergic neurons. The degeneration of these neurons in Parkinson's disease has recently been associated with SOD1 misfolding and dysfunction,[^13^, ^166b^] and diminished SOD1 antioxidant activity augments MPTP‐induced SNc dopamine neuron loss in animal models of Parkinson's disease.^[182]^

Aside from direct oxidative stress‐induced neuronal damage, the disruption of redox homeostasis within vulnerable neuronal populations may also contribute to neuron death by exacerbating other detrimental cellular pathologies, including Aβ plaque formation in Alzheimer's disease[^167a^, ^167b^] and α‐synuclein deposition in Parkinson's disease.^[166b]^ In this way, SOD1 dysfunction may constitute a modulator of disease progression, rather than an initial trigger for neurodegeneration, in some neurodegenerative disorders.

### 8.2 Gain‐of‐Function

Compounding variable consequences from a SOD1 loss‐of‐function, misfolded SOD1 protein exhibits an as‐of‐yet undefined toxic gain‐of‐function, strongly associated with motor neuron death in *SOD1*‐linked familial ALS. Over‐expression of human SOD1 mutants elicits severe motor neuron degeneration and a robust ALS phenotype in rodents^[156]^ and *D. rerio*,^[183]^ irrespective of superoxide dismutase activity in these animals. Similarly, knock‐in of ALS‐causing *SOD1* point mutations into the endogenous *Sod1* locus in *D. melanogaster* produces motor dysfunction and neurodegeneration without mutant protein over‐expression.^[184]^ High levels of soluble misfolded mutant SOD1 species and larger detergent‐insoluble mutant SOD1 protein aggregates accumulate selectively in neuronal regions most affected by the ALS disease process, indicating SOD1 self‐assembly is associated with severe motor neuron degeneration.[^13^, ^156^] Accordingly, when mutant protein expression is low and SOD1 accumulation is absent, ALS motor symptomology is not observed.^[185]^ Mounting evidence from post‐mortem patient tissues suggests a similar gain‐of‐function of misfolded wild‐type SOD1 may contribute to neurodegeneration in sporadic ALS,[^90b^, ^165a^, ^186^] idiopathic Parkinson's disease,^[13]^ and potentially Alzheimer's disease,^[91]^ although large insoluble misfolded SOD1 deposits are not consistently identified in patient tissues between studies.[^90a^, ^90b^] Variation in choice of immunodetection antibodies and methodologies and variation in type and quality of postmortem tissues between research groups likely contributes to the lack of reproducibility of these data. Titration of primary antibody dilutions and the consistency of staining protocols and primary antibodies employed between research groups could be improved. False‐positives may also arise in control tissues; harsher antigen retrieval protocols conceivably elicit synthetic SOD1 conformations, and metal‐chelating buffers (EDTA) disrupt SOD1 metalation to interfere with proper folding. These discrepancies could be reconciled through greater collaboration and the development and adoption of standardised methodological approaches. Substantial inconsistency further arises upon the use of “misfolded” SOD1 antibodies (C4F6, SEDI, EDI, B8H10, A5C3)—hydrophobic domain‐targeted primary antibodies recognizing SOD1 in non‐native conformations—whereby it is unclear whether immunopositivity signals immature unfolded protein, mature misfolded protein or a miriad of endogenous or synthetic folding states in between. Again, the creation and utilisation of validated, non‐denaturing immunodetection methods would likely greatly reduce variation between studies employing these antibodies, as would the completion of a thorough examination of the protein folding state(s) actually recognised by these antibodies.

In spite of these methodological considerations it is becoming increasingly clear that the toxicity of misfolded wild‐type and mutant SOD1 is independent of larger insoluble deposits, which are increasingly shown to be protective by mitigating the toxic effects of smaller oligomeric species.^[187]^ Rather, smaller oligomeric assemblages confer greater toxicity; pathologically affected tissues in murine models of *SOD1*‐linked familial ALS are enriched in soluble misfolded SOD1 prior to disease onset, whereas SOD1 aggregates increase markedly after symptom onset.^[188]^ In hSOD1^G93A^ mice, CNS regions least affected by the disease process have the most aggregated SOD1, and levels of aggregated SOD1 in the degenerating spinal cord are inversely correlated with disease progression.^[187b]^ These findings were corroborated in vitro by Brotherton and colleagues,^[189]^ whereby soluble misfolded SOD1 was toxic to cultured CHO cells and increasing aggregated SOD1 solubility enhanced cellular toxicity. Curiously, despite this connection between soluble misfolded SOD1 and cellular toxicity, levels of soluble misfolded SOD1 were found to be marginally decreased at end stage disease in transgenic mutant SOD1 mice.^[190]^ Whether this is a direct reflection of the severity of motor neuron loss, or of the distinct biochemistry of surviving neurons and glia, at this late disease stage remains to be determined. Traditional immunohistochemical approaches are unlikely to accuractely resolve smaller misfolded SOD1 species or assemblages, thus future investigations should consider alternative approaches (e.g. immunocapture or size‐based filtration)^[187b]^ to characterise smaller soluble misfolded SOD1 species in these disorders.

**Figure 10:**
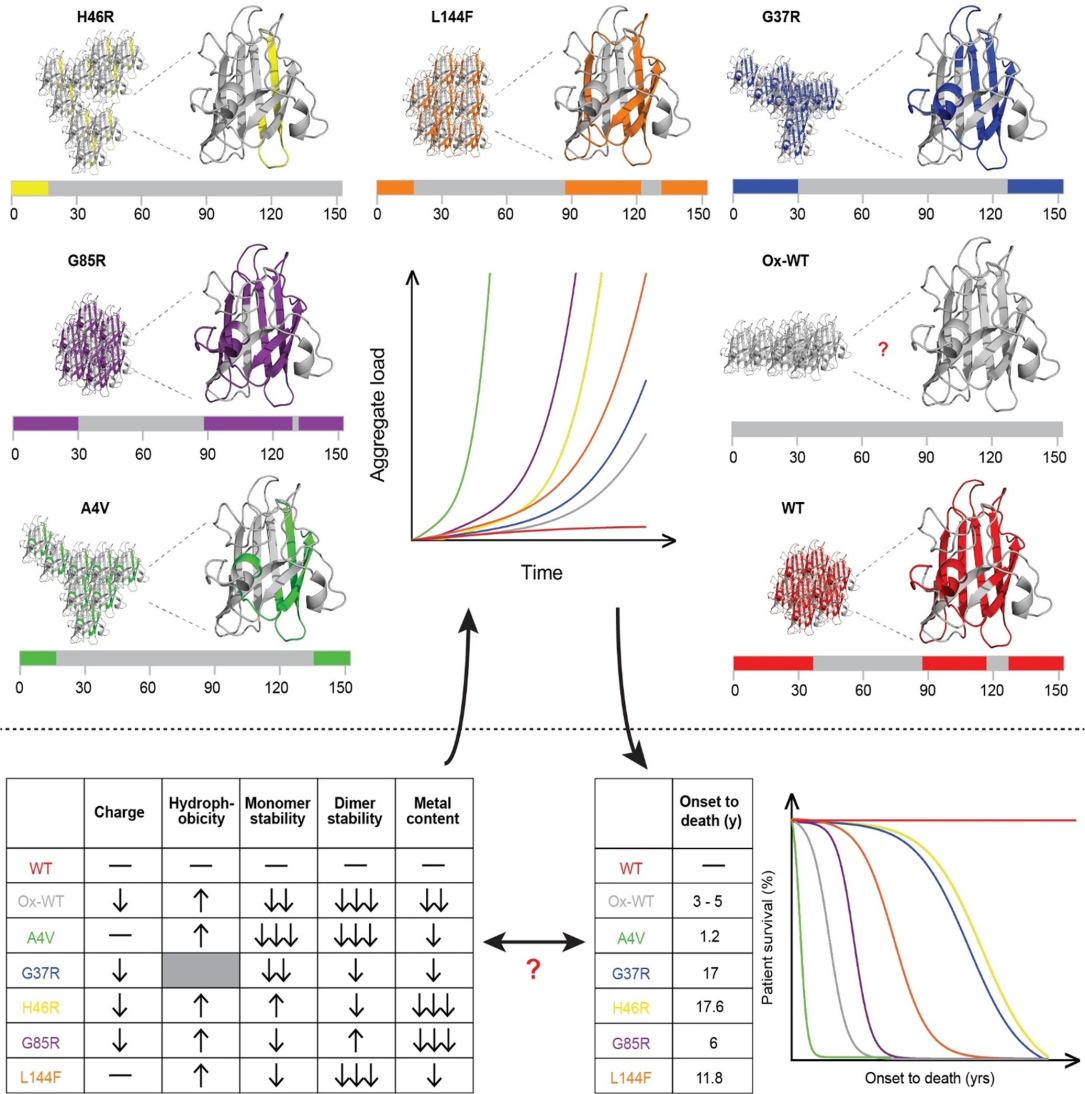
Heterogeneity in mechanisms of mutant and wild‐type SOD1 aggregation derives from differences in the biochemistry of soluble forms of each misfolded species, and may partially underlie variation in disease duration (onset to death) between individual *SOD1*‐linked familial ALS and sporadic ALS patients. Data was obtained from Table 2 in this review, grey shaded boxes represent no data available. Oxidised wild‐type SOD1 (Ox‐WT) onset to death (years) represented by average sporadic ALS disease duration. Coloured regions of grey bars represent residues of SOD1 protein implicated in self‐assembly for that misfolded variant, confirmed by mass spectrometry as reported by Furukawa and colleagues^[1]^ (specific residue numbers listed Supplementary Table 2).

The exact mechanisms of SOD1 self‐assembly appear to differ between misfolded species; SOD1 aggregates identified in post‐mortem *SOD1*‐linked familial ALS^[90a]^ and sporadic ALS^[90b]^ spinal cord, and Parkinson's disease brain,^[13]^ exhibit substantial morphological variability, and the aggregation rates of different SOD1 mutants fluctuate considerably (Table 2). Biochemical changes elicited by a given destabilizing factor (*SOD1* mutations, chemical modification of amino acid side chains) likely preference aggregation through one of multiple competing pathways, which derive from multiple aggregation‐prone protein sequences (Figure 10).^[1]^ Numerous sequences within the C‐terminus (101–107; 147–153),[^1^, ^191^] and β‐barrel (1–30; 28–38)[^1^, ^125^, ^192^] of SOD1 protein are key mediators of aggregation; however, their involvement in the self‐assembly of several mutant SOD1 variants is highly heterogeneous.[^1^, ^193^] Residues 90–120, for example, are implicated in the aggregation of wild‐type, G85R, G93R and L144F apo proteins, whereas residues 135–153 contribute to apo wild‐type, A4V, G37R G41D/S, G85R and G93A/R self‐assembly. Further, comprehensive epitope mapping of the entire human SOD1 protein sequence in hSOD1^G93A^, hSOD1^G85R^, hSOD1^D90A^, and hSOD1^WT^ mice demonstrated that multiple, structurally distinct SOD1 aggregates may coexist in vivo,^[193]^ indicating a coalescence of distinct aggregation pathways. In this study, changes in the relative abundances of two divergent aggregate strains closely resembled distinct patterns of ALS‐like clinical decline, suggesting that variability in the rate and structure of SOD1 aggregation could underlie phenotypic variability between *SOD1*‐linked familial ALS patients with different *SOD1* mutations (Figure 10).

In agreement, a recent investigation by Wang and colleagues^[100]^ applied a recalibrated Chiti‐Dobson equation to the most extensive set of patient data and mutant biophysical measurements to date, and found that 69 % of variability in the survival time of *SOD1*‐linked familial ALS patients could be accounted for by integrating the effects of *SOD1* mutations on aggregation rate and protein stability. These findings have not been completely recapitulated by other groups; however, similar trends have been identified.[^101d^, ^194^] Whilst this is a strong correlation, it is important to recognise that many biophysical measurements utilised in these studies originated from non‐human model systems, and yet are being correlated with human patient survival times. Considering there are substantial disparities in disease severity between humans and mice for a given SOD1 mutant, it is possible that a more accurate relationship between patient survival times and changes in SOD1 biochemistry may be generated by correlating biophysical measurements and indices of cellular viability within the same model systems. It is also important to acknowledge that, whilst patient survival data utilised in this study were comprehensive, these data were generated from small (*n*<5) patient cohorts for a significant number of mutations (29 of the 58 reported in Table 2). Bolstering datasets for these presumably rarer mutants will be an important step towards improving the accuracy and prognostic power of patient data, including age of onset and disease duration. Taking another approach, Alemasov and colleagues^[195]^ recently characterised alterations in hydrogen bonding networks in 35 mutant SOD1 proteins using a molecular dynamics simulation with elastic network modelling. Using these measurements they constructed a regression model for estimating the survival time of *SOD1*‐linked familial ALS patients, which exhibited a 0.91 correlation coefficient with measured patient survival times. Any remaining variability in *SOD1*‐linked familial ALS patient survival time may derive from non‐genetic contributors to SOD1 misfolding, from compounding environmental or genetic influences differing between patients, or from inaccurate patient data. Identifying relationships between non‐genetic destabilizing factors and specific pathways of aggregation constitutes an important area for further investigation, and may underlie variability in the progression of *SOD1*‐linked familial ALS, as well as neurodegenerative disorders characterised by wild‐type SOD1 misfolding, such as sporadic ALS and Parkinson's disease.

**Figure 11:**
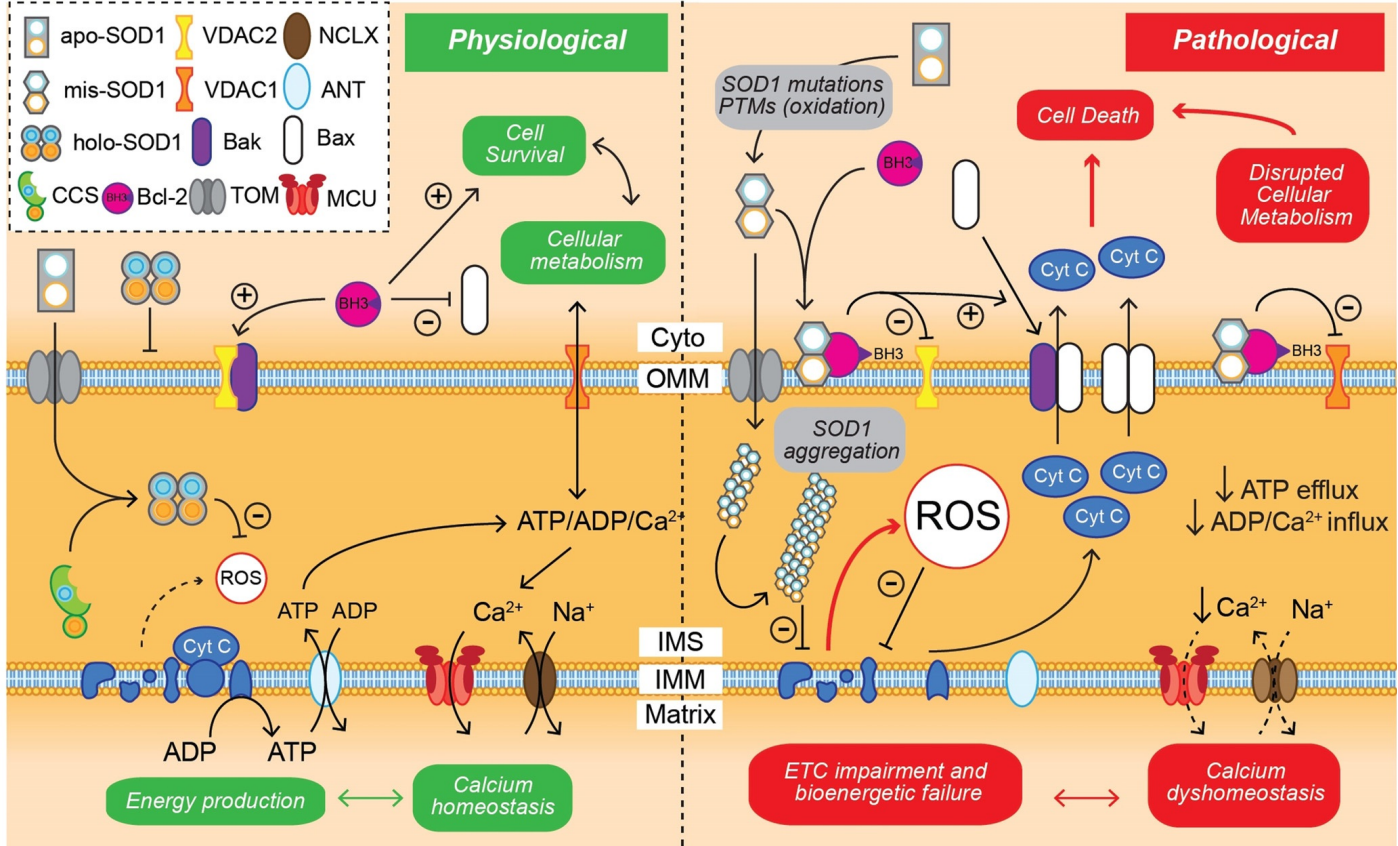
Mitochondrial SOD1 biology under physiological and pathological conditions. In a healthy cell apo‐SOD1 is transferred from the cytosol (Cyto) into the mitochondrial intermembrane space (IMS) by translocase of the outer membrane (TOM). Cu chaperone for SOD1 (CCS) facilitates metal insertion and maturation of apo‐SOD1 to holo‐SOD1 in the IMS, which detoxifies reactive oxygen species (ROS) produced by the electron transport chain (ETC) in the inner mitochondrial membrane (IMM). ATP produced by the ETC is essential for maintaining many cellular metabolic processes, and is shuttled between the mitochondrial matrix and the cytosol by adenine nucleotide translocator (ANT) and voltage‐dependent anion‐selective channel 1 (VDAC1), located in the IMM and outer mitochondrial membrane (OMM), respectively. VDAC1 is also crucial for ADP and Ca^2+^ import, which together drive mitochondrial energy production. Calcium may be stored in the mitochondrial matrix, with fluxes across the IMM controlled by mitochondrial calcium uniporter (MCU) and the mitochondrial sodium calcium exchanger NCLX. *SOD1* gene mutations or atypical post‐translational modifications to SOD1 protein result in misfolded SOD1 (mis‐SOD1) protein, which either remains in the cytosol or enters the mitochondrial IMS, where it is shown to aggregate. Aggregated SOD1 is unable to detoxify ROS in the IMS, and is proposed to impair ETC function to promote the accumulation of ROS and pro‐apoptotic cytochrome c (Cyt C) in this compartment. Cytosolic mis‐SOD1 binds to Bcl‐2, an anti‐apoptotic protein which normally inhibits the function of pro‐apoptotic proteins Bax and Bak. SOD1 binding causes conformational change in Bcl‐2, exposing its toxic BH3 domain. SOD1‐Bcl‐2‐BH3 complexes localise to the OMM where they inhibit VDAC1 and promote the formation of Bax and Bak pores. Together, these actions disrupt mitochondrial calcium homeostasis and ATP production, and facilitate the release of Cyt C into the cytosol, triggering cell death.

Multiple molecular pathways may underlie the association between SOD1 self‐assembly and neurotoxicity. As discussed previously, the toxicity of misfolded SOD1 likely derives from smaller soluble species, which possess a greater sub‐cellular accessibility into vital organelles, especially mitochondria. Accordingly, one pathway of neurotoxicity hypothesised to result from smaller misfolded SOD1 species involves the release of mitochondrial cytochrome c and the activation of caspase‐dependent programmed cell death (Figure 11). These events are proposed to be triggered by the accumulation of misfolded mutant SOD1 oligomers on the outer mitochondrial membrane, reported in vitro (G93A, G85R)^[196]^ and in mutant SOD1 murine models (G93A, G37R).^[197]^ Outer mitochondrial membrane localisation is highly Bcl‐2‐dependent in *SOD1*‐linked familial ALS patients, mutant SOD1 murine models and cell culture.^[198]^ Under physiological conditions, Bcl‐2 impedes the release of pro‐apoptotic factors from mitochondria, including cytochrome c, preventing caspase activation and apoptosis.^[199]^ Misfolded SOD1 binding, however, triggers conformational change in Bcl‐2 that exposes its toxic BH3 domain, triggering cytochrome c release and apoptosis.[^197b^, ^198^] Further, misfolded SOD1/Bcl‐2 complexes are suggested to reduce outer mitochondrial membrane permeability by directly inhibiting voltage‐dependent anion channel 1,^[200]^ an outer mitochondrial membrane porin regulating mitochondrial ATP production and export. This may contribute to bioenergetic failure, increased ROS production, and calcium dyshomeostasis within motor neurons of pre‐symptomatic G93A mice,^[201]^ associated with ALS pathogenesis in these animals. Mutation of the BH3 domain of Bcl‐2 prevents mutant SOD1 mitochondrial toxicity in vitro,^[198]^ indicating Bcl‐2 may represent a novel therapeutic target for ameliorating mutant SOD1 toxicity in *SOD1*‐linked familial ALS. Importantly, oxidised wild‐type SOD1 recapitulates this toxic interaction,^[165b]^ suggesting common pathogenic mechanisms and therapeutic targets between *SOD1*‐linked familial ALS and disorders exhibiting oxidised wild‐type SOD1, including sporadic ALS,[^90b^, ^165b^] Parkinson's disease[^13^, ^91^] and Alzheimer's disease.^[91]^

Similar to its accumulation on the outer mitochondrial membrane, misfolded SOD1 targeted to the mitochondrial inter‐membrane space results in motor neuron toxicity in mutant SOD1 cell culture and transgenic mice.^[202]^ Apo‐SOD1 enters the mitochondrial intermembrane space via translocase of the outer mitochondrial membrane in the outer mitochondrial membrane, and subsequent CCS‐mediated metal incorporation and disulfide bond formation trap SOD1 within this compartment (Figure 6).^[76]^ If SOD1 in the intermembrane space becomes misfolded, substantial SOD1 aggregation may occur within this compartment, associated with electron transport chain dysfunction and mitochondrial ROS production (Figure 11).^[203]^ To examine mechanisms underlying mutant SOD1‐mediated mitochondrial dysfunction a number of studies co‐expressed human CCS with mutant SOD1 in cell cultures and mice, promoting misfolded mutant SOD1 accumulation within the intermembrane space.[^158^, ^202a^] CCS overexpression itself did not elicit an abnormal mitochondrial or behavioural phenotype; however, mutant SOD1‐linked ALS symptomology was markedly more enhanced in double transgenic mice compared with regular mutant SOD1 mice. Neuron death was associated with reduced electron transport chain function, particularly mitochondrial cytochrome c oxidase,^[158]^ which was proposed to derive from the preferential delivery of Cu to human SOD1 at the expense of cytochrome c oxidase. Supplementation of culture media with Cu sulfate failed to restore mitochondrial respiratory function in hSOD1^G93A^‐transfected NSC‐34 cells;^[203b]^ however, treatment of hSOD1^G93A^/hCCS mice with the orally bioavailable Cu delivery agent diacetylbis(N(4)‐methylthiosemicarbazonato) Cu^II^(Cu^II^(atsm)) restored cytochrome c oxidase activity and significantly extended survival time,^[154]^ suggesting Cu‐dependent mechanism(s) of mitochondrial dysfunction driven by misfolded SOD1 may indeed be present. Importantly, Cu^II^(atsm) treatment improves Cu delivery to SOD1 in mutant SOD1 transgenic mice and improves survival time despite eliciting an increase in the total amount of mutant SOD1 protein present,^[151]^ suggesting SOD1‐mediated mitochondrial dysfunction is closely tied to SOD1 Cu metalation rather than the amount of SOD1 protein. In addition to SOD1 Cu metalation, in vitro data generated from hSOD1^G93A^‐transfected NSC‐34 cells implicates nitric oxide (NO) accumulation in SOD1‐associated mitochondrial dysfunction,^[204]^ with substantial improvements in electron transport chain function elicited by NO scavengers.^[203b]^ It is unclear whether misfolded SOD1 toxicity similarly derives from NO production in vivo; however, as reducing NO accumulation via pharmacological inhibition of neuronal NO synthase, or genetic ablation of inducible NO synthase, failed to improve survival rates of mutant SOD1 transgenic mice.^[98b]^ Cu^II^(atsm) is known to mitigate NO toxicity,^[205]^ and it is possible that this mechanism of action contributes to its therapeutic efficacy in mutant SOD1 transgenic mice, although further investigations are warranted. Mutant SOD1‐mediated NO toxicity is believed to result from Zn‐deficient SOD1;^[204]^ however, misfolded SOD1 mutant proteins are reported to be Cu‐deficient in murine models in vivo,[^151^, ^154^] and no evidence exists for Zn‐deficient SOD1 protein in patient or murine model tissues.

In contrast to *SOD1* mutations, little is known regarding the impact of atypical post‐translational modifications on misfolded wild‐type SOD1‐mediated mitochondrial dysfunction. Given that mitochondrial SOD1 import and localisation is highly dependent upon the presence of specific post‐translational modifications, including disulfide bond formation, acylation,^[206]^ and potentially palmitolylation, investigations into the consequences of atypical post‐translational modifications for SOD1 mitochondrial localisation and cellular toxicity are warranted.

### 8.3 One Protein, Multiple Phenotypes: A Concept of Neuronal Vulnerability

Aside from mitochondrial dysfunction, misfolded SOD1 is implicated in the initiation and/or acceleration of numerous damaging pathways in both neurons[^13^, ^59^, ^207^] and surrounding glia,^[208]^ including disruption of proteasome function, degradation of microtubules and microfilaments, endoplasmic reticulum stress, and redox dyshomeostasis. These pathways are evident in regions exhibiting severe neuronal degeneration in ALS, Parkinson's disease and Alzheimer's disease, and thus misfolded SOD1 may contribute to the initiation and/or progression of one or more of these pathologies in each disorder, contributing to neuronal damage or death. The idea that misfolded or dysfunctional SOD1 may play a role in the degeneration of three such functionally and anatomically disparate neuronal populations is an underexplored possibility consistent with the notion that diverse neurodegenerative conditions may share common underlying mechanisms of disease. Consistent with this hypothesis are data demonstrating that restoring or bolstering physiological SOD1 structure and function is associated with higher neuronal densities in vulnerable populations, and longer survival times, in animal models of all three disorders.[^151^, ^154^, ^182^, ^209^]

We recently proposed that biochemical similarities between vulnerable neuronal populations in ALS and Parkinson's disease promote the evolution of a shared detrimental biomolecular cascade, within which misfolded SOD1 constitutes a key therapeutic target.^[166b]^ Spinal cord motor neurons^[210]^ and SNc dopamine neurons are prone to mild redox dyshomeostasis, mitochondrial dysfunction and protein misfolding during healthy aging,^[166b]^ which become progressively exacerbated by additional pathogenic factors such as genetic mutations, biometal dysregulation (Cu, Fe) or exposure to toxins. Such intrinsic biochemical characteristics are likely to predispose these neurons, over other neuronal populations, to SOD1 misfolding during respective disease pathogeneses. Despite many valuable insights gained from studying the consequences of *SOD1* mutations, we argue for a greater focus to be directed towards non‐genetic factors underlying SOD1 misfolding, which may contribute to the highly selective pattern of neurodegeneration in both disorders. In *SOD1*‐linked familial ALS patients, mutant SOD1 is expressed ubiquitously throughout *SOD1*‐linked familial ALS patients, yet mutant SOD1 misfolding and toxicity manifests selectively within upper and lower motor neurons in these patients, and very rarely occurs before mid‐way through their fourth decade of life.^[211]^ In Parkinson's disease patients the accumulation of misfolded SOD1 protein in the vulnerable SNc occurs in the absence of known SOD1 gene mutations.^[166a]^ Together these data suggest that non‐genetic factors are important contributors to the disease process in both disorders. The identification of non‐genetic factors underlying region‐specific SOD1 misfolding and deposition in *SOD1*‐linked familial ALS, which may also contribute to wild‐type SOD1 misfolding in sporadic ALS, idiopathic Parkinson's disease and potentially Alzheimer's disease will significantly improve our understanding of the biology of this protein. Further, it will be particularly important to develop our understanding of glia‐derived SOD1 toxicity in these disorders, with data from *SOD1*‐linked familial ALS patients providing clear evidence for a fundamental role of misfolded SOD1‐mediated glial dysfunction in the selective vulnerability of motor neurons. Overall we propose that the manifestation of a shared neurodegenerative pathway as multiple distinct phenotypes can arise from the degeneration of specific and distinct neuronal populations in each disease, resulting in differing disease onsets, symptoms, and durations, depending on the cell populations most affected.

## 9 Summary and Outlook

Therapeutic translation of our rapidly progressing aetiological knowledge of all major neurodegenerative disorders remains disappointingly slow. There are no clinically available treatments capable of slowing or halting the progression of neurodegeneration in Parkinson's or Alzheimer's disease, and whilst two drugs have been approved for the treatment of ALS, both offer limited clinical benefit. Riluzole affords ALS patients a 2–4 month improvement in survival time from symptom onset, although this occurs without a verifiable improvement in motor functional impairment^[212]^ and may therefore prolong life during disease stages where quality of life is poorest.^[213]^ Edaravone yields a significant (33 %) functional improvement in ALS patients as measured by the revised ALS Functional Rating Scale,^[214]^ although it is unclear how this impacts patient survival time overall. Beyond a rudimentary understanding of their ability to reduce oxidative stress and cytotoxicity, our knowledge of any neuroprotective mechanisms mediated by Riluzole and Edaravone is virtually absent. Moreover, their limited efficacy suggests they do not target key drivers of neurodegeneration. Identifying and selectively targeting key components of neurodegenerative disease pathways represents the best strategy to halt or slow the progression of neurodegeneration in ALS and other complex neurodegenerative disorders.

Multiple compounds with reported effects on SOD1 function and deposition are currently underway in clinical trials for *SOD1*‐linked familial ALS and sporadic ALS (Table 3), following improvements in survival and motor function in mutant SOD1 murine models of ALS. Mechanistically, these therapies minimise SOD1 toxicity by either restoring physiological SOD1 structure and function, or by reducing the total amount of SOD1 protein available to misfold and assemble. IONIS SOD1Rx and pyrimethamine constitute two examples of the latter currently being trialled in the clinic,[^209a^, ^215^] with other RNAi‐based approaches such as AAV9‐SOD1‐shRNA not far behind.^[216]^ Whilst these therapies exhibit potential in ameliorating the ALS phenotype,[^215^, ^217^] the removal of SOD1 protein may predispose neurons to degeneration by alternative mechanisms in the longer term, as observed in *Sod1* ^*−/−*^ mice.^[89]^ Co‐administration of a SOD mimetic along with IONIS SOD1Rx or pyrimethamine may partially address this concern, although to date no such combinational approaches have been investigated. GC4711 and GC4419 constitute two potential candidate SOD mimetics developed to reduce radiation‐induced severe oral mucositis in cancer patients, whose safety and tolerability are currently being tested in phase I clinical trials in two separate healthy cohorts (NCT03099824, NCT03762031). Aside from appropriate combinational approaches, we propose that ideal monotherapies should stabilise misfolded SOD1 to simultaneously ameliorate toxic molecular interactions and restore optimal catalytic function, rather than completely remove a protein with such an important role in maintaining neuron health.

**Table 3:** Summary of clinical trials with reported effects on SOD1 function and/or misfolding in neurodegenerative diseases. Status/Results are as recorded on 10 January 2020 at https://clinicaltrials.gov.

Authors	Study Design	Participants	SOD1 Biology	Outcome Measures	Status/Results
Galera Therapeutics Inc. (NCT03762031)	Phase I, DPRCT	40 healthy participants (aged 18–50)	GC4711—SOD mimetic, catalyzes superoxide dismutation	Number of participants with treatment‐emergent adverse events and/or laboratory abnormalities	Recruiting
Galera Therapeutics Inc. (NCT03099824)	Phase I, non‐randomized, open‐label clinical trial	60 healthy participants (aged 18–50)	GC4711, GC4419—SOD mimetics, catalyze superoxide dismutation	Number of participants with treatment‐emergent adverse events and/or laboratory abnormalities	Recruiting
Weill Medical College of Cornell University^[207]^ (NCT01083667)	Phase I/II, multi‐centre, open label clinical trial	32 familial ALS patients with confirmed *SOD1* gene mutations	Pyrimethamine—reduction in SOD1 protein production	Mean Change in SOD1 CSF	Complete—significant reduction in CSF SOD1 protein
Ionis Pharmaceuticals Inc.^[201a]^ (NCT01041222)	Phase I, DPRCT	33 familial ALS patients with confirmed *SOD1* gene mutations	IONIS SOD1Rx—antisense oligonucleotide targeted to SOD1 mRNA, promotes mRNA degradation	Number of participants with treatment‐emergent adverse events and/or laboratory abnormalities, CSF and plasma SOD1 protein levels	Complete—no serious adverse events, dose‐dependent CSF and plasma concentrations observed
Biogen Inc. and Ionis Pharmaceuticals Inc. (NCT02623699)	Phase I, multi‐centre, DPRCT	84 familial ALS patients with confirmed *SOD1* gene mutations	IONIS SOD1Rx—as above	As above	Active, not recruiting
Biogen Inc. and Ionis Pharmaceuticals Inc. (NCT03070119)	Phase I, non‐randomized, open‐label clinical trial	48 familial ALS patients with confirmed *SOD1* gene mutations, must have completed Part A and/or Part B of study NCT02623699	IONIS SOD1Rx—as above	As above	Enrolling by invitation
University of Miami^[212]^ (NCT00706147)	Phase II/III DPRCT	38 familial ALS patients with confirmed *SOD1* gene mutations	Arimoclomol—promotes heat shock protein‐dependent regulation of SOD1 protein folding, reduces misfolding	Safety and tolerability, and preliminary efficacy (ALSFRS‐R, FEV6, CAFS)	Complete—safe and well‐tolerated for up to 12 months. Possible therapeutic benefit.
Collaborative Medicinal Development Pty Limited (NCT02870634)	Phase I, multi‐centre, open‐label clinical trial	50 familial/sporadic ALS patients	Cu^II^(atsm)—increases SOD1 protein Cu binding and catalytic activity, and reduces mutant SOD1 toxicity.	Safety and tolerability, RP2D, preliminary efficacy (ALSFRS‐R), drug pharmacokinetics (plasma levels)	Active, not recruiting
Collaborative Medicinal Development Pty Limited (NCT03136809)	Phase I/II, multi‐centre, open label, treatment extension study	50 familial/sporadic ALS patients, must have completed 6 month assessment in study NCT02870634	Cu^II^(atsm)—as above	Tolerance of extended treatment, preliminary efficacy (ALSFRS‐R)	Active, not recruiting
Collaborative Medicinal Development Pty Limited (NCT04082832)	Phase II/III, multi‐centre, DPRCT	80 familial/sporadic ALS patients	Cu^II^(atsm)—as above	ALSFRS‐R, ECAS, seated slow vital capacity, frequency of adverse events	Recruiting
Collaborative Medicinal Development Pty Limited (NCT03204929)	Phase I, multi‐centre, open label clinical trial	38 early Parkinson's disease patients, within 5 years of clinical diagnosis, H&R stage ≤2.	Cu^II^(atsm)—as above	RP2D, UPDRS	Active, not recruiting

ALSFRS‐R, Revised ALS Functional Rating Scale; CAFS, Combined Assessment of Function and Survival; CSF, cerebrospinal fluid; DPRCT, double‐blind placebo‐controlled randomized clinical trial; ECAS, Edinburgh Cognitive and Behavioral Amyotrophic Lateral Sclerosis Screen; FEV6, percent predicted forced expiratory volume in 6 seconds; H&R, Hoehn and Yahr scale; IV, intravenous; RP2D, recommended phase II dose; UPDRS, Unified Parkinson's disease rating scale. Further clinical trial information available in Table 3 in the Supporting Information.

One such example, Arimoclomol, induces heat shock protein responses,^[218]^ which promote the natural folding of nascent proteins and refolding of misfolded proteins.^[219]^ Administration of Arimoclomol resulted in improved motor function and motor neuron survival in G93A mutant SOD1 mice,^[218]^ and recent data from a phase II clinical trial demonstrates it is safe and tolerable at a therapeutic dose (up to 300 mg day^−1^) for a short period of time (12 weeks) in patients with ALS.^[220]^ Unfortunately, comprehensive biochemical analyses of SOD1 activity, protein levels and metal content have not been performed in Arimoclomol‐treated G93A mice, and thus any relationship between therapeutic benefit and modulation of misfolded mutant SOD1 can only be inferred. The cysteine‐reactive selenium‐based compound Ebselen likewise facilitates SOD1 maturation by assisting the formation of the stabilizing intramolecular SOD1 disulfide bond, significantly reducing mutant SOD1 aggregation in vitro and in vivo in mutant SOD1 mice.^[221]^ Further experiments are warranted to characterise the impact of Ebselen on the antioxidant capacity and viability of vulnerable neuronal populations in these models.

The apparent promise of Ebselen in reducing SOD1 accumulation in vivo hints at the therapeutic potential of modulating post‐translational modification of SOD1 protein to counteract SOD1 misfolding, suggesting a greater research focus be paid towards developing similar treatment strategies. Pharmacological interaction partners of SOD1′s Trp32 residue, for example, have the potential to lessen oxidation‐induced SOD1 misfolding and aggregation, and may prevent SOD1 toxicity.[^110^, ^222^] Amongst these, a series of aromatic compounds exploit hydrophobic interactions with the Trp32 indole ring system, and four catecholamines are found to interact with a grove in the SOD1 surface close to Trp32 created by loop II.^[223]^ In addition to Trp32, cysteinylation of Cys111 blocks oxidation or glutathionylation of this residue's indole side chain and exerts a comparatively negligible destabilizing effect on SOD1 protein, protecting against oxidative stress‐induced SOD1 aggregation.[^110^, ^224^] Sumoylation of Lys75 increases mutant SOD1 protein stability and promotes the formation of large insoluble aggregates, preventing the accumulation of smaller toxic misfolded species.^[225]^ Phosphorylation of, or phospho‐mimetic modifications to, Thr2 within the dimer interface counteracts structural changes elicited by certain *SOD1* mutations (A4V) via unknown mechanisms,[^120b^, ^226^] stabilizing mutant SOD1 and reducing neuronal toxicity in vitro. Aspirin‐induced acetylation of three or more lysine residues within human SOD1 impairs mutation‐induced SOD1 fibrillation in vitro (A4V),^[227]^ suggesting that interventions which increase the net negative surface charge of SOD1 protein may attenuate self‐assembly. When considering how to best utilise these beneficial modifications in a therapeutic setting, fundamental difficulties will foreseeably arise in directing such ubiquitous cellular modifications to specific residues of a target protein. Accordingly, successful therapeutic strategies in this area should aim to harness endogenous regulatory machinery governing these pathways, the caveat being that we first need to adequately understand their underlying molecular bases and consequences.

In addition to modulating the post‐translational modifications discussed above, improving metal delivery to SOD1 is reported to yield the greatest therapeutic benefit to multiple strains of mutant SOD1 transgenic mice to date.[^151^, ^154^, ^228^] This is consistent with data demonstrating that the toxicity of misfolded mutant SOD1 protein is more closely associated with SOD1 metal content than the total amount of mutant SOD1 protein in these mice.^[151]^ The pharmacological agent employed in all of these studies, Cu^II^(atsm), specifically delivers Cu^I^ to regions exhibiting oxidative stress, hypoxia, and mitochondrial electron transport chain impairment,[^228^, ^229^] where it is reported to remetalate aggregation‐prone, Cu‐deficient SOD1 and improve SOD1 catalytic activity.[^151^, ^154^, ^228^] In all instances, a diminution in Cu‐deficient SOD1 in the vulnerable spinal cord was accompanied by improvements in motor neuron density, motor function and mouse survival, suggesting an association between SOD1 Cu metalation and motor neuron degeneration. If administered to hSOD1^G93A^/hCCS mice during the pre‐symptomatic disease phase Cu^II^(atsm) prevented the development of ALS altogether,^[154]^ whilst administration to hSOD1^G93A^ and hSOD1^G37R^ mice after symptom onset markedly improved locomotor function and survival time.[^228^, ^230^] Importantly, the therapeutic benefit of this compound across numerous mutant SOD1 transgenic mouse strains has been independently verified by multiple research groups.[^154^, ^231^] Accordingly, phase II/III clinical trials of Cu^II^(atsm) have recently begun in a small cohort of ALS patients (*n=*80; Table 3). Given that perturbations to SOD1 metalation appear to be restricted to the central nervous system in mammalian models of ALS,^[157]^ no outcome measures for any of the current clinical trials of Cu^II^(atsm) in familial and sporadic ALS patients involve measurement of SOD1 metalation, protein levels or catalytic activity, and thus any association between therapeutic benefit and alterations to SOD1 biochemistry can only be inferred from these trials. Furthermore, Cu^II^(atsm) treatment reportedly mitigates nitrosative stress^[205]^ and neuroinflammation^[232]^ in vivo, and promotes neurite elongation in vitro,^[233]^ suggesting other neuroprotective actions of Cu^II^(atsm) in ALS that are largely, if not completely, independent of SOD1. It therefore must be acknowledged that any therapeutic efficacy identified for Cu^II^(atsm) in the treatment of ALS may also feasibly occur via pathways that are either in conjunction with, or entirely independent of, alterations to SOD1 biochemistry.

The identification of Cu^II^(atsm) as a highly effective combatant against nitrosative stress galvanised interest in its possible use a treatment for Parkinson's disease, where nitrosative stress is implicated in multiple degenerative pathways including α‐synuclein toxicity, DNA oxidation and lipid peroxidation.^[234]^ Cu^II^(atsm) treatment ameliorated dopaminergic neuron loss, rescued dopamine metabolism, improved motor function and prolonged survival time in multiple murine models of Parkinson's disease, and was also able to prevent peroxynitrate‐driven toxicity in differentiated human neuroblastoma SH‐SY5Y cells and reduce peroxynitrate‐induced aggregation of α‐synuclein in vitro.^[205]^ A phase I clinical trial of Cu^II^(atsm) was subsequently initiated in a small cohort of Parkinson's disease patients (Table 3). The recent discovery of misfolded wild‐type SOD1 deposition in the vulnerable SNc of Parkinson's disease patients[^13^, ^166a^] indicates that modulation of SOD1 biochemistry may partially underlie the putative therapeutic efficacy of Cu^II^(atsm) in Parkinson's disease animal models, similar to that observed in mutant SOD1 transgenic mice. Direct characterisation of SOD1 protein levels, function and structure are warranted in the vulnerable SNc of these animals before and after Cu^II^(atsm) treatment to identify SOD1 as a therapeutic target of Cu^II^(atsm), as was performed in *SOD1*‐linked familial ALS murine models. Similar to current clinical trials of Cu^II^(atsm) in ALS, there are no outcome measures quantifying SOD1 metalation, protein levels or catalytic activity in the current trial of Cu^II^(atsm) in Parkinson's disease due to the likely restriction of SOD1 mismetalation to central nervous system tissue. However, if feasible, such measurements would again be able to inform on the relationship between modulation of SOD1 biochemistry and therapeutic efficacy in Parkinson's disease patients.

Most significantly, the association of misfolded and dysfunctional SOD1 protein with dopaminergic neuron loss in Parkinson's disease suggests that many of the therapies currently being trialled for their ability to influence misfolded mutant SOD1 biochemistry in *SOD1*‐linked familial ALS (Table 3) may also constitute novel disease‐modifying treatments for Parkinson's disease. Such an idea opens remarkable possibilities for the efficient translation of treatments between distinct neurodegenerative disorders which exhibit SOD1 misfolding and dysfunction.

## Conflict of interest

The authors declare no conflict of interest.

## Biographical Information

*Benjamin Trist received his BSc(Adv) with Class 1 Hons (Pharmacology) in 2014 from the University of New South Wales (Australia) and his PhD (Medicine) in 2019 from the University of Sydney. He has since been working as a postdoctoral researcher at the Brain and Mind Centre at the University of Sydney. His research focuses on the role of biometals and oxidative stress in protein misfolding and neuron death in neurodegenerative movement disorders, focussing on Parkinson's disease and amyotrophic lateral sclerosis*.

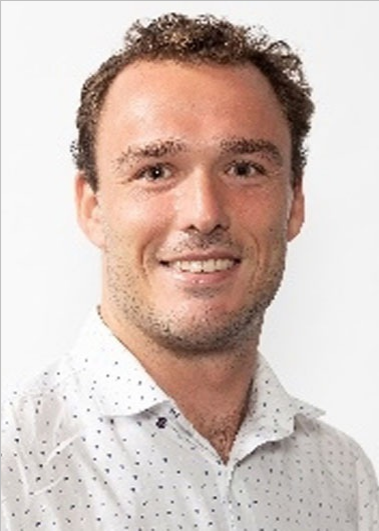

## Biographical Information

*James Hilton obtained his BSc Hons degree in Science majoring in pharmacology and neuroscience at the University of Melbourne (Australia). He completed his PhD in pathology from the University of Melbourne in 2017. He currently holds the MNDRIA Beryl Bayley MND Postdoctoral Fellowship at the University of Melbourne in the Department of Pharmacology and Therapeutics. His research interest focuses on metallobiology in neurodegeneration and targeting these pathways therapeutically*.

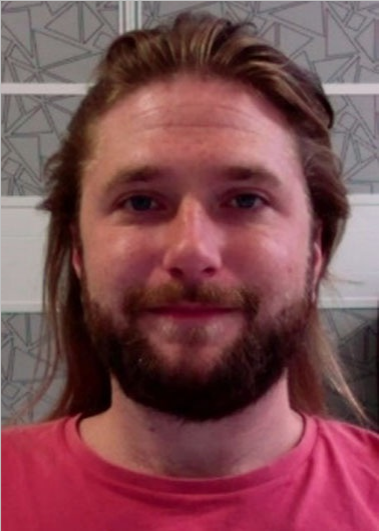

## Biographical Information

*Dominic Hare is an applied chemist with a PhD from the University of Technology Sydney, Australia (2009). He worked with Distinguished Prof. Philip Doble at UTS developing biological imaging techniques in mass spectrometry before joining the Florey Institute of Neuroscience and Mental Health (Australia) in 2012. In 2020 he moved to the School of BioSciences at the University of Melbourne as Associate Professor to establish a dedicated bioanalytical R&D laboratory with support from Agilent Technologies aimed at developing new analytical technologies for clinical research*.

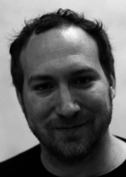

## Biographical Information

*Peter Crouch trained as a plant biochemist, obtaining his PhD from La Trobe University (Australia) in 2002. He transitioned to neuroscience research at the commencement of his first postdoctoral appointment. He is currently an Associate Professor at the University of Melbourne, where his laboratory focuses on the elemental basis of neurodegeneration and elucidating pathways amenable to pharmacological intervention*.

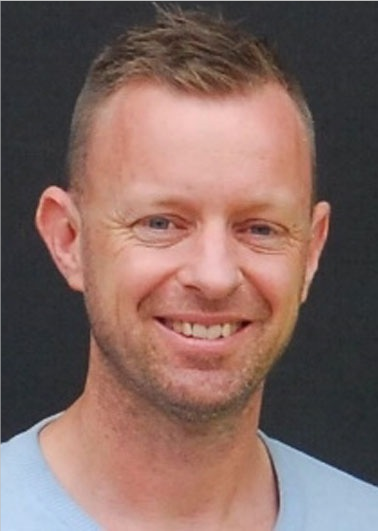

## Biographical Information

*Kay Double is a Professor of Neuroscience at the Brain and Mind Centre at the University of Sydney (Australia). Kay holds a PhD (1993, Australia) and a habilitation (2005, Germany) in neurochemistry. Her work focuses on cellular pathways underlying neuronal vulnerability in neurodegenerative disorders of movement and dementia, primarily using human tissues. She has a particular interest in biometals and metalloproteins. Her ultimate aim is to develop disease‐modifying interventions which slow or halt disease processes*.

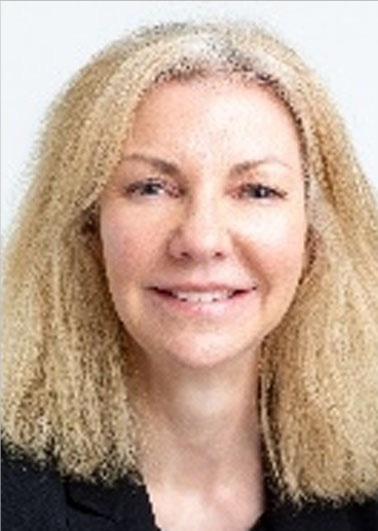

## Supporting information

As a service to our authors and readers, this journal provides supporting information supplied by the authors. Such materials are peer reviewed and may be re‐organized for online delivery, but are not copy‐edited or typeset. Technical support issues arising from supporting information (other than missing files) should be addressed to the authors.

Supplementary
