# Supplementary material for: Superoxide Dismutase 1 in Health and Disease: How a Frontline Antioxidant Becomes Neurotoxic
Source: Angew Chem Int Ed Engl. 2020 Nov 19;60(17):9215–46. doi: 10.1002/anie.202000451 (PMC8247289; doi:10.1002/anie.202000451)
Supplement: Supplementary file 1 — Supplementary [file ANIE-60-9215-s001.pdf]

Angewandte Chemie
Eine Zeitschrift der Gesellschaft Deutscher Chemiker

Supporting Information

Superoxide Dismutase 1 in Health and Disease: How a Frontline Antioxidant Becomes Neurotoxic

Benjamin G. Trist, James B. Hilton, Dominic J. Hare, Peter J. Crouch, and Kay L. Double**

anie_202000451_sm_miscellaneous_information.pdf

Author Contributions

B.T. Conceptualization: Lead; Writing - Original Draft: Lead; Writing - Review & Editing: Equal
J.H. Writing - Review & Editing: Equal
P.C. Writing - Review & Editing: Equal
D.H. Writing - Review & Editing: Equal
K.D. Conceptualization: Supporting; Supervision: Lead; Writing - Review & Editing: Equal.

Supplementary Information

Supplementary Table 1. Specific amino acid residues contained within key structural features of human SOD1 protein, as well as those constituting sites of post-translational modification. Sites of post-translational modification were identified from analyses of human SOD1 protein using mass spectrometry (PhosphoSitePlus®, Cell Signalling Technology), with the exception of carbonyl oxidation, which were identified *in silico*.

Structural Feature	***Residues***
Electrostatic loop	121-142
Metal binding loop	49-85
Greek key loop	102-115
Disulfide loop	49-62
Disulfide bond	57, 146
Copper-binding residues	46, 48, 63, 120
Zinc-binding residues	63, 71, 80, 83
Electrostatic loop - charged residues	121, 122, 124, 125, 128, 132, 133, 136
Electrostatic loop - polar residues	131, 134, 135, 137, 139, 142
Dimer interface residues	5, 7, 17, 50-54, 113-115, 148, 150-153
Post-translational Modification	***Residues***
Oxidation (carbonylation)	2, 3, 6, 9, 54, 62, 111, 115, 116
Oxidation (cysteinic acid)	111
Oxidation (oxo-histidine)	46, 48, 71, 80, 120
Oxidation (tryptophan)	32
Glutathionylation	111
Phosphorylation	2, 25, 34, 58, 59, 68, 88, 98, 102, 105, 107, 39
Acetylation	9, 23, 70, 122
Succinylation	9, 75, 122
Ubiquitylation	3, 9, 23, 30, 36, 70, 75, 91, 122, 128, 136
Glycation	3, 9, 30, 36, 122, 128
Palmitolyation	6, 57, 111, 146
Deamidation	26, 131, 139
Methylgloxalation	69, 79, 143

Supplementary Table 2. Specific amino acid residues implicated in the self-assembly of multiple mutant SOD1 variants and wild-type SOD1 protein. Residues confirmed by mass spectrometry, previously reported by Furukawa and colleagues.

Variant	***Residues***
Wild Type	1-37, 87-125, 127-153
A4V	1-16, 135-153
G37R	1-30, 127-153
H46R	1-16
G85R	1-30, 87-135, 137-153
L144F	1-16, 87-122, 137-153

Supplementary Table 3. Full summary of clinical trials with reported effects on SOD1 function and/or misfolding in neurodegenerative diseases. Status/Results are as recorded on the 10th January 2020 at https://clinicaltrials.gov.

Authors	Study Design	Participants	Intervention/Duration	SOD1 Biology	Outcome Measures	Status/Results
Galera Therapeutics Inc. (NCT03762031)	Phase I, DPRCT	40 healthy participants (aged 18-50)	• Single dose intervention<br>• Dose escalation<br>• Randomization<br>1. GC4711 (30mg)<br>2. GC4711 (60mg)<br>3. GC4711 (90mg)<br>4. GC4711 (120mg)<br>5. Placebo	GC4711 – SOD mimetic, catalyzes superoxide dismutation	Number of participants with treatment-emergent adverse events and/or laboratory abnormalities	Recruiting
Galera Therapeutics Inc. (NCT03099824)	Phase I, non-randomized, open-label clinical trial	60 healthy participants (aged 18-50)	• Single dose intervention<br>• Dose escalation<br>• Randomization<br>1. IV GC4419 (27mg)/ GC4711 Oral Capsule G-101 (82mg)<br>2. IV GC4419 (27mg)/ GC4711 Oral Capsule G-101 (164mg)<br>3. IV GC4419 (27mg)/ GC4711 Oral Capsule G-101 (246mg)<br>4. IV GC4419 (27mg)/ GC4711 Oral Capsule G-111 (175mg)<br>5. IV GC4419 (27mg)/ GC4711 Oral Capsule G-112 (145mg)<br>6. IV GC4419 (30mg)/ GC4711 Oral Capsule G-119 (233mg)<br>7. IV GC4419 (30mg)/ GC4711 Oral Capsule G-125 (233mg)	GC4711, GC4419 – SOD mimetics, catalyze superoxide dismutation	Number of participants with treatment-emergent adverse events and/or laboratory abnormalities	Recruiting
Weill Medical College of Cornell University[207] (NCT01083667)	Phase I/II, multi-centre, open label clinical trial	32 familial ALS patients with confirmed *SOD1* gene mutations	• 9 month intervention<br>• Single arm - dose escalation to 75mg/day if tolerated	Pyrimethamine – reduction in SOD1 protein production	Mean Change in SOD1 CSF	Complete – significant reduction in CSF SOD1 protein
Ionis Pharmaceuticals	Phase I, DPRCT	33 familial ALS patients with	• Single intrathecal infusion - 12hrs<br>• 28 day follow-up period<br>• Randomization (8 per cohort; 6 drug, 2 placebo)	IONIS SOD1Rx – antisense oligonucleotide targeted to	Number of participants with treatment-emergent adverse events and/or	Complete – no serious adverse events, dose-dependent CSF

Inc.[201a] (NCT01041222)		confirmed *SOD1* gene mutations	1. IONIS SOD1Rx (0.15mg)<br>2. IONIS SOD1Rx (0.5mg)<br>3. IONIS SOD1Rx (1.5mg)<br>4. IONIS SOD1Rx (3.0mg)	SOD1 mRNA, promotes mRNA degradation	laboratory abnormalities, CSF and plasma SOD1 protein levels	and plasma concentrations observed
Biogen Inc. and Ionis Pharmaceuticals Inc. (NCT02623699)	Phase I, multi-centre, DPRCT	84 familial ALS patients with confirmed *SOD1* gene mutations	• 24 week intervention<br>• Randomization (doses not specified, drug and placebo within each cohort)<br>1. Single ascending dose<br>2. Multiple ascending dose	IONIS SOD1Rx – as above	As above	Active, not recruiting
Biogen Inc. and Ionis Pharmaceuticals Inc. (NCT03070119)	Phase I, non-randomized, open-label clinical trial	48 familial ALS patients with confirmed *SOD1* gene mutations, must have completed Part A and/or Part B of study NCT02623699	• 12 month intervention<br>• Randomization (doses not specified)<br>1. Low dose<br>2. Medium dose<br>3. High dose A<br>4. High dose B	IONIS SOD1Rx – as above	As above	Enrolling by invitation
University of Miami[212] (NCT00706147)	Phase II/III DPRCT	38 familial ALS patients with confirmed *SOD1* gene mutations	• 12 month intervention<br>• Randomized<br>1. Arimoclomol (600mg/day)<br>2. Placebo	Arimoclomol – promotes heat shock protein-dependent regulation of SOD1 protein folding, reduces misfolding	Safety and tolerability, and preliminary efficacy (ALSFRS-R, FEV6, CAFS)	Complete - safe and well-tolerated for up to 12 months. Possible therapeutic benefit.
Collaborative Medicinal Development Pty Limited (NCT02870634)	Phase I, multi-centre, open-label clinical trial	50 familial/sporadic ALS patients	• 6 month intervention<br>• Dose escalation (1 month, dose cohorts, n=6, 3-48mg/day)<br>• Dose expansion (1 - 6 months, subject to eligibility, RP2D)	Cu^{II}(atsm) – increases SOD1 protein Cu binding and catalytic activity, and reduces mutant SOD1 toxicity.	Safety and tolerability, RP2D, preliminary efficacy (ALSFRS-R), drug pharmacokinetics (plasma levels)	Active, not recruiting

Collaborative Medicinal Development Pty Limited (NCT03136809)	Phase I/II, multi-centre, open label, treatment extension study	50 familial/sporadic ALS patients, must have completed 6 month assessment in study NCT02870634	• 24 month intervention<br>• Single arm - RP2D	Cu^{II}(atsm) – as above	Tolerance of extended treatment, preliminary efficacy (ALSFRS-R)	Active, not recruiting
Collaborative Medicinal Development Pty Limited (NCT04082832)	Phase II/III, multi-centre, DPRCT	80 familial/sporadic ALS patients	• 24 week intervention (6x4 week cycles)<br>• Randomized<br>1.Cu^{II}(atsm) (72mg/day)<br>2.Placebo	Cu^{II}(atsm) – as above	ALSFRS-R, ECAS, seated slow vital capacity, frequency of adverse events	Recruiting
Collaborative Medicinal Development Pty Limited (NCT03204929)	Phase I, multi-centre, open label clinical trial	38 early Parkinson's disease patients, within 5 years of clinical diagnosis, H&R stage ≤ 2.	• 6 month intervention<br>• Dose escalation (1 month, dose cohorts, n=6, ≥12mg/day)<br>• Dose expansion (1 - 6 months, n=20 patients, subject to eligibility, RP2D)	Cu^{II}(atsm) – as above	RP2D, UPDRS	Active, not recruiting

Abbreviations - ALSFRS-R, Revised ALS Functional Rating Scale; CAFS, Combined Assessment of Function and Survival; CSF, cerebrospinal fluid; DPRCT, double-blind placebo-controlled randomized clinical trial; ECAS, Edinburgh Cognitive and Behavioral Amyotrophic Lateral Sclerosis Screen; FEV6, percent predicted forced expiratory volume in 6 seconds; H&R, Hoehn and Yahr scale; IV, intravenous; RP2D, recommended phase II dose; UPDRS, Unified Parkinson's disease rating scale.
